# High-Entropy Amorphous Catalysts for Water Electrolysis: A New Frontier

**DOI:** 10.1007/s40820-025-01936-5

**Published:** 2025-10-20

**Authors:** Gaihong Wang, Zhijie Chen, Jinliang Zhu, Jiangzhou Xie, Wei Wei, Yi-Ming Yan, Bing-Jie Ni

**Affiliations:** 1 Centre for Technology in Water and Wastewater, School of Civil and Environmental Engineering, University of Technology Sydney, Sydney, NSW 2007 Australia; 2 UNSW Water Research Centre, School of Civil and Environmental Engineering, The University New South Wales, Sydney, NSW 2052 Australia; 3 School of Resources, Environment and Materials, State Key Laboratory of Featured Metal Materials and Life-Cycle Safety for Composite Structures, Guangxi University, Nanning, 530004 People’s Republic of China; 4 School of Mechanical and Manufacturing Engineering, University of New South Wales, Sydney, NSW 2052 Australia; 5 State Key Lab of Organic−Inorganic Composites, Beijing Advanced Innovation Center for Soft Matter Science and Engineering, Beijing University of Chemical Technology, Beijing, 100029 People’s Republic of China

**Keywords:** High‐entropy amorphous catalysts, Electrocatalysis, Water splitting, Structural disorder, Multimetallic synergy

## Abstract

High‐entropy amorphous catalysts (HEACs) integrate multielement synergy with structural disorder, making them promising candidates for water splitting. Their distinctive features—including flexible coordination environments, tunable electronic structures, abundant unsaturated active sites, and dynamic structural reassembly—collectively enhance electrochemical activity and durability under operating conditions. This review summarizes recent advances in HEACs for hydrogen evolution, oxygen evolution, and overall water splitting, highlighting their disorder-driven advantages over crystalline counterparts. Catalytic performance benchmarks are presented, and mechanistic insights are discussed, focusing on how multimetallic synergy, amorphization effect, and in‐situ reconstruction cooperatively regulate reaction pathways. These insights provide guidance for the rational design of next‐generation amorphous high‐entropy electrocatalysts with improved efficiency and durability.

## Highlights

- This review comprehensively summarizes the recent progress of high-entropy amorphous catalysts for electrochemical water splitting.
- The unique structural characteristics of high-entropy amorphous materials—such as short-range order, high defect density, and flexible coordination—are discussed in relation to their electrocatalytic advantages.
- Mechanistic insights into multimetallic synergy, amorphization effect, and in-situ reconstruction are highlighted to guide rational catalyst design.

## Graphical abstract

**Figure:**
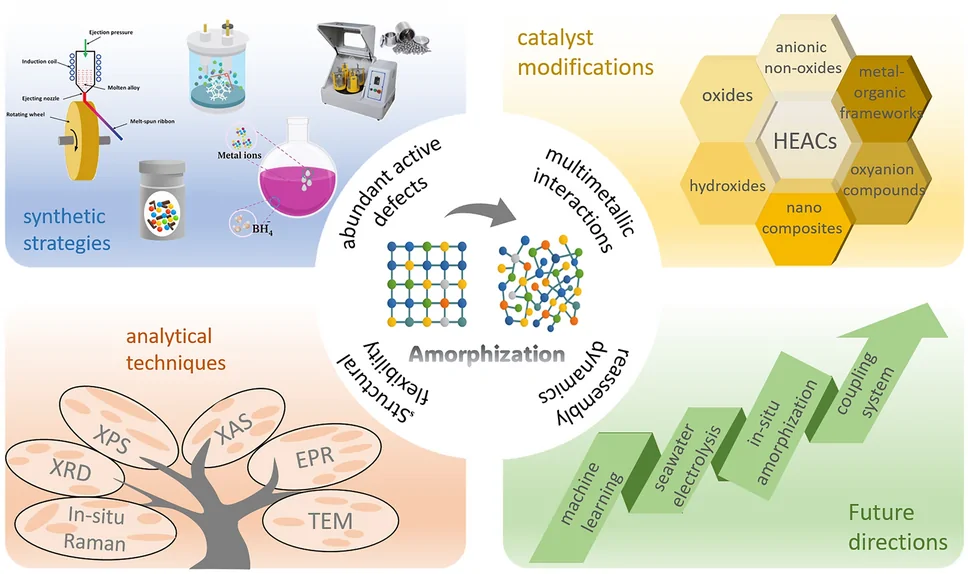

## Introduction

The urgent global demand for sustainable energy technologies has placed green hydrogen at the forefront of next-generation fuel strategies [1, 2]. Among the various production routes, water electrolysis—powered by renewable electricity—offers a clean and scalable approach to hydrogen generation [3, 4]. However, the sluggish kinetics of the hydrogen evolution reaction (HER) and, more critically, the oxygen evolution reaction (OER) impose significant overpotentials and efficiency losses, thereby necessitating the development of efficient, stable, and cost-effective electrocatalysts [5, 6]. Despite decades of research, noble metal-based catalysts such as Pt and Ir/Ru oxides remain benchmarks for HER and OER, respectively, but their scarcity and high cost hinder large-scale deployment [7, 8]. This has motivated intense exploration of alternative catalyst systems capable of delivering comparable performance while meeting practical criteria for scalability, durability, and compositional tunability.

In this context, high-entropy materials (HEMs) have emerged as an exciting class of catalytic materials that leverage multicomponent elemental design to unlock new structure–property landscapes [9, 10]. A central thermodynamic principle underlying the stability of HEMs is the concept of high configurational entropy [11, 12]. Unlike conventional alloys that are typically composed of one or two principal elements, HEMs consist of five or more elements in near-equimolar ratios [13, 14]. This multicomponent configuration dramatically increases the system’s configurational entropy, which plays a decisive role in stabilizing single-phase solid solution structures, particularly at elevated temperatures. According to the Gibbs free energy equation [15, 16]:

1 $$\Delta {{G}} = \Delta {{H}} - {{T}}\Delta {{S}}$$

an increase in entropy (ΔS) effectively lowers the Gibbs free energy (ΔG), favoring the formation of thermodynamically stable solid solutions rather than intermetallic or phase-separated structures. For an ideal equimolar n-component system, the configurational entropy can be estimated by [17, 18]:

2 $${\Delta S}_{\mathrm{config}} = - {{R}}\mathop \sum \limits_{i = 1}^{n} c_{i} \ln c_{i}$$

where *R* is the gas constant and *c* is the atomic fraction of each component. In a five-component equimolar system, this yields Δ*S* ≈ 1.61R, exceeding the empirical threshold (1.5R) often used to classify materials as high-entropy systems.

This elevated entropy contributes not only to phase stabilization but also expands the design space for novel material architectures [19–21]. It enables the formation of solid solutions even among elements with substantial differences in atomic size, electronegativity, or bonding preference [22, 23]. Additionally, lattice distortion arising from atomic size mismatches induces local strain and electronic perturbations, thereby enhancing the adsorption of reaction intermediates [9, 24]. The presence of multiple diffusion barriers leads to sluggish atomic diffusion, which in turn improves the structural stability under reaction conditions [25, 26]. Moreover, the multielement synergy derived from the cocktail effect enables tunable catalytic activity and durability that surpass those of single-element systems [27, 28].

**Fig. 1:**
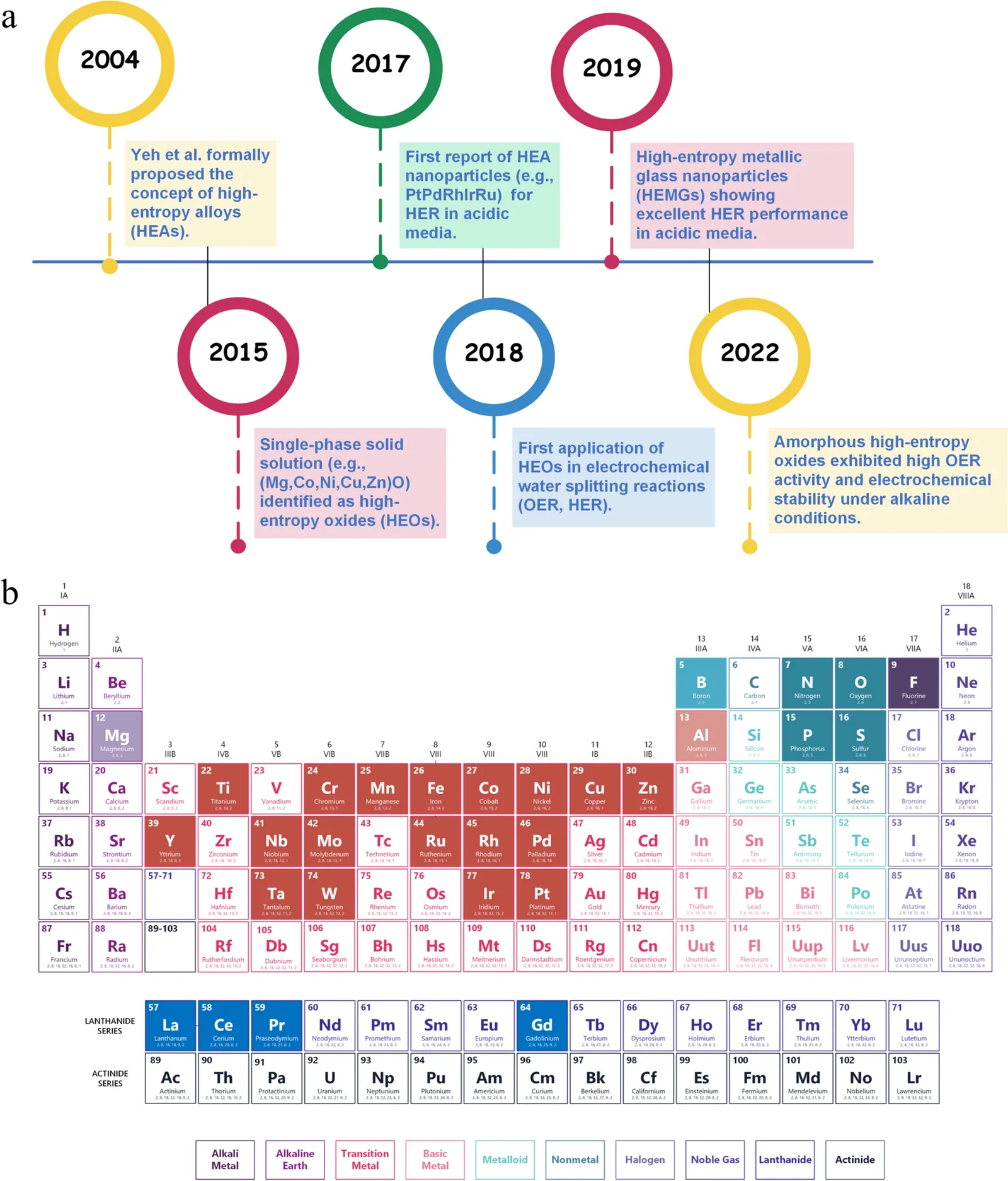
Progress and elemental trends in high-entropy materials. **a** Historical timeline of the development of high-entropy materials in application of water splitting. **b** Highlighted elements frequently incorporated in high-entropy electrocatalysts for HER and OER

This concept was first introduced by Yeh et al., who proposed the design of high-entropy alloys (HEAs) using multiple principal metallic elements (Fig. 1a) [12]. Initially developed for structural applications, HEAs were later extended into functional domains, culminating in the introduction of high-entropy oxides (HEOs) in 2015 by Rost et al. [29]. The prototypical HEO (Mg, Co, Ni, Cu, Zn)O displayed remarkable phase stability, compositional flexibility, and electronic tunability, opening a new avenue for catalytic applications under electrochemical environments. The electrocatalytic potential of high-entropy systems rapidly gained momentum thereafter. In 2017, HEA nanoparticles composed of Pt, Pd, Rh, Ir, and Ru were demonstrated to exhibit outstanding HER performance in acidic media [30]. In 2018, HEOs were further explored for both HER and OER catalysis [31, 32]. In these systems, the choice of constituent elements plays a pivotal role in dictating both the amorphous structure and catalytic activity (Fig. 1b). 3d transition metals such as Fe, Co, Ni, Mn, Cr, Cu, and Zn are frequently employed due to their abundant redox states and cost-effectiveness [33, 34]. These are often combined with 4d/5d noble and semi-noble metals (e.g., Mo, W, Ru, Ir) or rare earth elements (e.g., Ce, La) to modulate conductivity, electronic structure, and corrosion resistance [35, 36]. Furthermore, anion incorporation (e.g., P, S, B) enables the formation of compound classes such as high-entropy phosphides, sulfides, and nitrides, offering new avenues for surface polarity tuning and intermediate adsorption [37, 38]. Parallel to the development of crystalline high-entropy systems, a growing body of research has highlighted the amorphization of catalysts as a transformative strategy for enhancing catalytic performance. By 2019, a new subclass of high-entropy metallic glasses was reported, combining high-entropy design with amorphous structure, which enabled rich undercoordinated sites and favorable electron transport properties for HER [39]. In 2022, amorphous high-entropy oxides emerged as effective OER electrocatalysts in alkaline environments, highlighting the role of structural disorder in facilitating active-site accessibility and long-term durability [40].

Amorphous structures are defined by their lack of long-range atomic order but exhibit short-range coordination, abundant defects, and dynamic atomic configurations [41, 42]. These features result in enhanced surface reactivity, isotropic ion/electron transport, and flexible electronic environments that are particularly advantageous for electrochemical reactions under harsh conditions [43–45]. When combined with the compositional complexity and synergistic effects of high-entropy design, high-entropy amorphous catalysts (HEACs) emerge as highly adaptable and robust catalysts, especially under extreme or dynamically changing conditions. Compared to their crystalline counterparts, HEACs present several unique advantages. The disordered atomic arrangement in amorphous structures eliminates crystal plane limitations and grain boundaries, allowing for the exposure of more unsaturated active sites [46, 47]. Additionally, the high density of defects and diverse local coordination environments facilitate electronic structure modulation and enable alternative reaction pathways, thereby enhancing catalytic selectivity and activity [48, 49]. The absence of lattice matching requirements also reduces interfacial strain during redox cycling, contributing to improved structural stability [50]. However, despite recent progress, the majority of high-entropy catalysts reported to date remain crystalline or partially crystalline. Systematic efforts toward the rational design, controllable synthesis, and mechanistic elucidation of HEACs are still at an early stage. In particular, fundamental questions persist regarding how atomic-scale disorder couples with entropy-stabilized multielement interactions, and how these complex systems evolve structurally and electronically under operating conditions.

To date, several reviews have focused on either high-entropy catalysts or amorphous electrocatalysts for water splitting, each highlighting their respective advantages in terms of active-site diversity, structural flexibility, and catalytic performance. However, a systematic review dedicated to the emerging class of amorphous high-entropy catalysts is still lacking. Accordingly, this review aims to provide a comprehensive and critical overview of recent progress in amorphous high-entropy catalysts for water electrolysis. We begin by introducing this concept and elaborating on their fundamental structural characteristics, highlighting their unique advantages in electrocatalysis compared to crystalline analogues. We then survey state-of-the-art synthetic strategies—including electrodeposition, hydrothermal/solvothermal methods, melt-spinning with dealloying, ball milling, and chemical reduction—emphasizing their roles in tailoring structural disorder, optimizing elemental distribution, and enhancing catalytic functionality. Next, we discuss the applications of high-entropy amorphous systems in HER, OER, and overall water splitting, with emphasis on activity metrics, long-term stability, and comparisons with benchmark materials. Finally, we explore mechanistic insights, with a focus on how multimetallic synergy, the amorphous effect, and in-situ reconstruction collectively govern reaction pathways. Building on these perspectives, we outline promising future directions, including accelerating the rational design of electrocatalysts through the integration of density functional theory and machine learning, exploring the hidden potential of amorphous high-entropy non-oxide anionic catalysts for seawater electrolysis, constructing in-situ amorphization-driven interfaces in high-entropy catalysts to enhance electrocatalytic performance, and coupling hydrogen and oxygen evolution reactions with value-added redox processes to develop integrated electrochemical systems.

## Structural Characteristics of High-Entropy Amorphous Catalysts

**Fig. 2:**
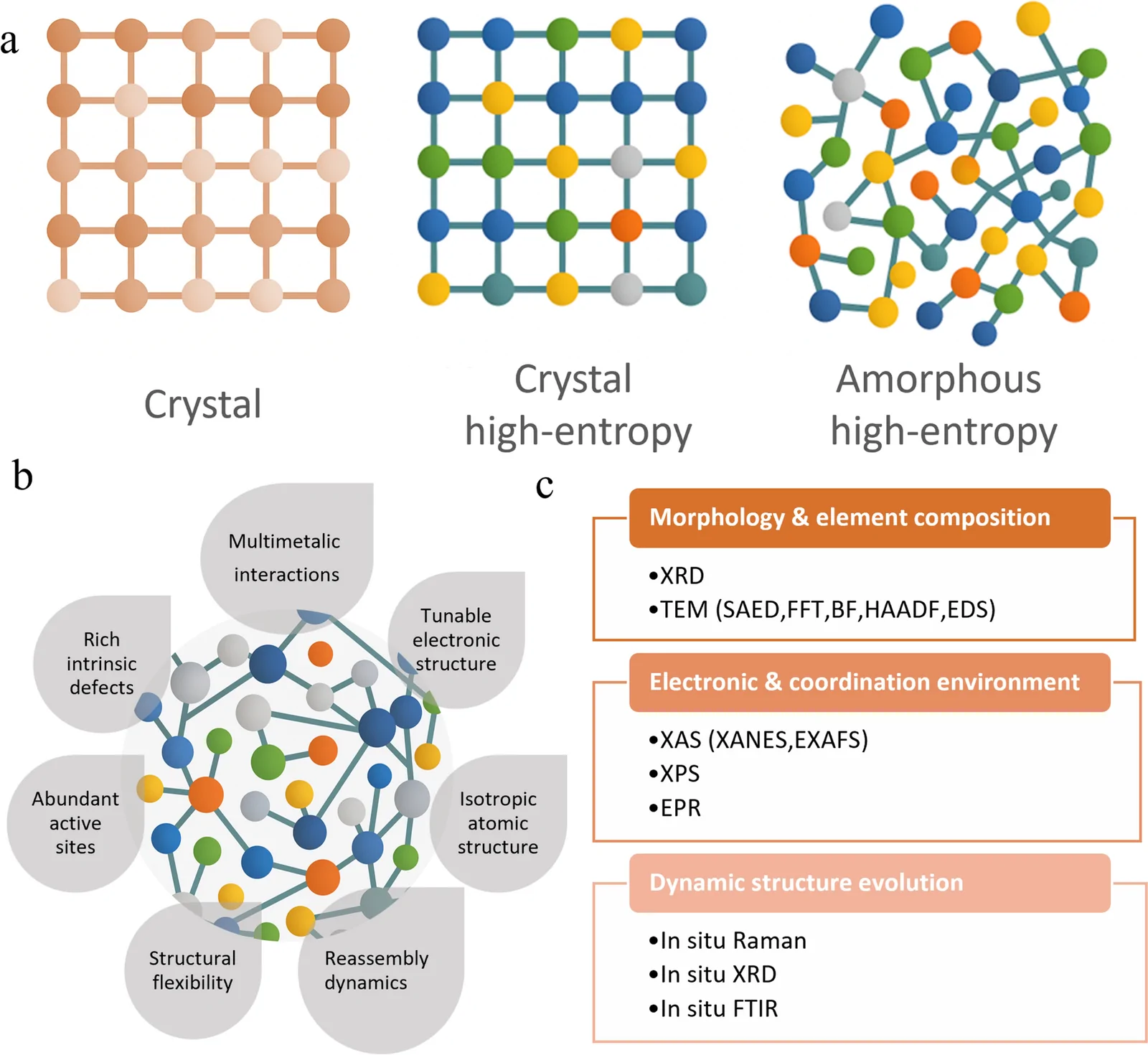
Overview of structure and characterization of HEACs. **a** Schematic diagram. **b** Structural characteristics. **c** Characterization techniques

HEACs derive their unique electrocatalytic properties from the synergistic combination of structural disorder and multielemental composition (Fig. 2a). Unlike their crystalline or single-metal counterparts, HEACs exhibit a range of intrinsic features—such as abundant defects, structural flexibility, tunable electronic structure and multicomponent active centers—that collectively enable enhanced activity, selectivity, and stability under complex electrochemical conditions (Fig. 2b). This section systematically outlines the key structural characteristics that govern the performance of HEACs, providing a foundation for understanding their catalytic behavior in water-splitting reactions.

### Abundant Active Defects

HEACs possess substantial structural disorder, which generates a high density of catalytically active defects such as vacancies, dangling bonds, undercoordinated metal sites, and interfacial boundariesm [51, 52]. These defects, often formed during rapid solidification, non-equilibrium growth, or reductive synthesis, not only provide abundant active centers but also modulate the local electronic environment. Furthermore, the absence of long-range order enables active sites to extend from the surface into the bulk, amplifying the overall catalytic contribution. For example, the FeCoNiMnRu-TA catalyst with a nanoflower-like architecture showed a dominant pore size of approximately 8.15 nm, promoting greater exposure of catalytic active sites [53]. The O1*s* X-ray photoelectron spectroscopy (XPS) spectrum also exhibited an intensified peak associated with defective oxygen, confirming the abundant structural defects in FeCoNiMnRu-TA that contributed to the generation of additional active sites. Moreover, FeCoNiMnBPOx exhibited the highest concentration of oxygen vacancies, which enhanced electrical conductivity and facilitated intermediate adsorption [54]. It also showed the largest ECSA value (50.875 cm^2^), indicating a greater exposure of active centers.

### Multimetallic Interactions

In HEACs, the coexistence of multiple principal metallic elements gives rise to pronounced multimetallic interactions, including orbital hybridization, charge redistribution, and local strain effects [55, 56]. These interactions break the uniformity of the electronic environment, generating a broad distribution of local coordination and oxidation states. As a consequence, the electronic structure—particularly the position of the d-band center and the density of states near the Fermi level—can be finely tuned. For example, XPS and ultraviolet photoelectron spectroscopy (UPS) analyses of Fe_20_Co_20_Ni_20_Mo_20_Al_20_ revealed that dealloying induced oxidation of Fe, Co, Ni, and Mo to higher valence states, while electron loss from Co atoms enriched the electron density around Ni atoms, indicating strengthened Ni–Co electronic coupling [57]. These synergistic electron transfers, combined with a d-band center shift and enhanced adsorption capability, were likely responsible for its improved catalytic activity.

### Structural Flexibility

The isotropic atomic arrangement in HEACs ensures uniform distribution of mechanical stress during electrochemical operation. More importantly, the absence of long-range periodicity imparts exceptional structural flexibility, allowing the atomic network to undergo local rearrangements without generating high-energy defects [58, 59]. This flexibility enables the material to accommodate substantial volume fluctuations during redox processes—particularly in gas-evolving reactions—while suppressing crack formation and particle detachment. As a result, HEACs can maintain their structural integrity and catalytic performance over prolonged cycling, even under high current densities. Feng et al. demonstrated the mechanical robustness and corrosion resistance of the spent catalyst after a 100-h stability test in alkaline seawater by examining its morphological and structural integrity [60]. The microflower architecture of HEO-FeCoNiMoVOx-1.5 remained intact even under high-magnification TEM image, showing no evidence of surface corrosion. TEM analysis further confirmed the absence of structural collapse, highlighting the catalyst’s exceptional structural durability.

### Reassembly Dynamics

The metastable nature of the amorphous phase in HEACs facilitates dynamic structural reassembly under catalytic operating conditions, often generating more active surface phases with enriched defect sites [61]. Meanwhile, the lattice distortion effect of high-entropy catalysts makes the atomic stacking of the system relatively loose, structurally disordered, and susceptible to surface reconstruction [62]. In addition, the inherently high surface energy and morphology plasticity of HEACs promote intimate and persistent contact with conductive substrates, thereby minimizing interfacial resistance and enabling efficient charge and mass transfer across the catalyst-substrate interface. The energy state can be assessed from the Δ*H* curves, obtained by integrating the heat capacity profiles. Cai et al. reported that FeCoNiCrMo_1_ exhibited the largest Δ*H* value (540–810 K), indicating its high-energy state [63]. Notably, amorphous materials with higher Δ*H* values typically possess larger free volumes, enabling their surfaces to adapt more readily to the shapes and sizes of adsorbate molecules, thereby enhancing adsorption.

### Characterization Techniques for Structural Analysis

**Fig. 3:**
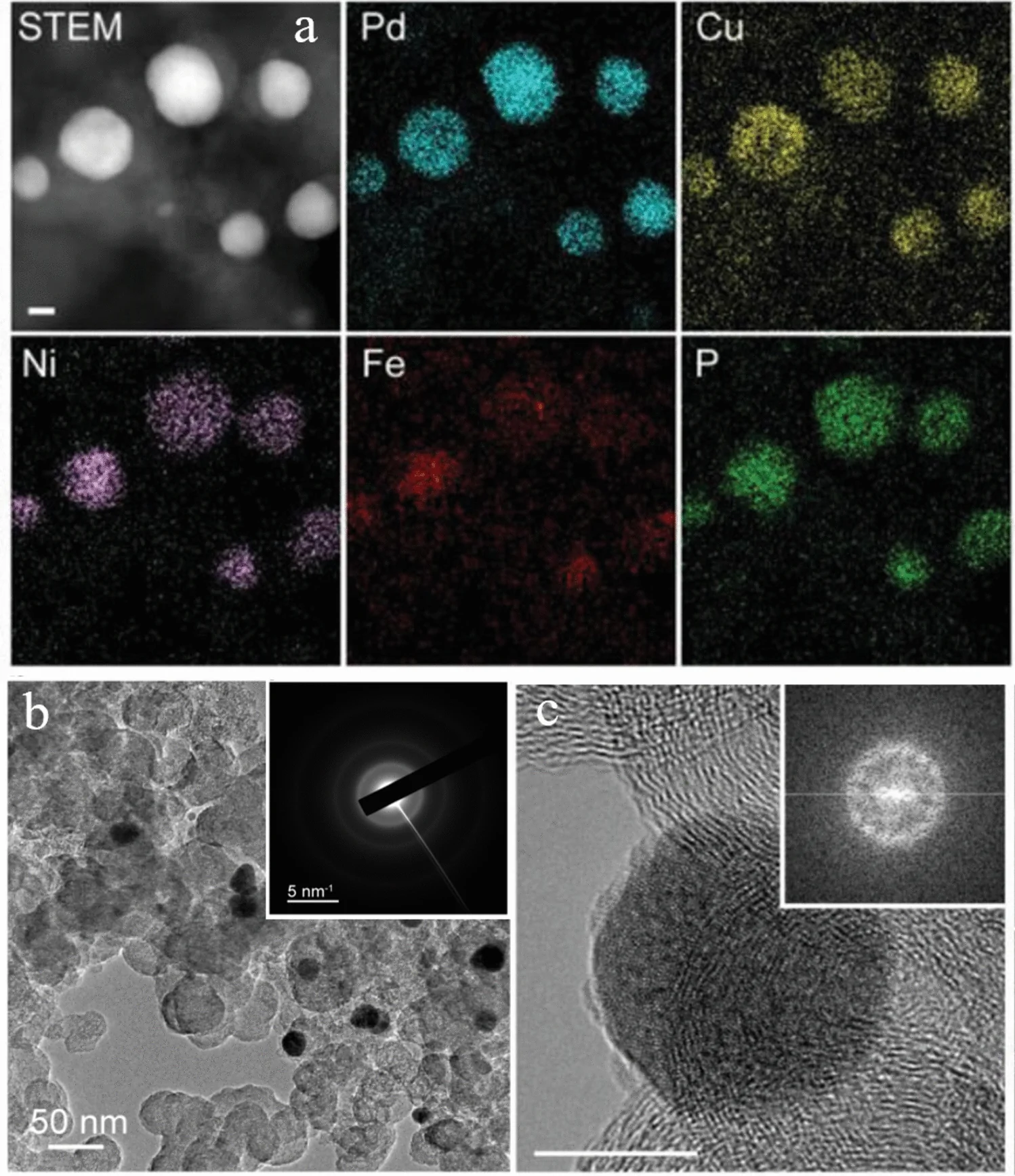
TEM characterization. **a** HADDF-STEM image and EDX elemental maps. **b** BF-TEM image with the inset of SAED pattern. The scale bar is 50 nm for the TEM image, and 5 nm^−1^ for the SAED pattern. **c** HRTEM image and the corresponding FFT image (inset). Scale bar, 5 nm. Reproduced with permission [66]. Copyright 2024, John Wiley and Sons

To unravel the complex structure–property relationships in HEACs, a comprehensive suite of characterization techniques is indispensable (Fig. 2c) [64]. These methods enable a multidimensional understanding of short-range atomic ordering, elemental distribution, electronic structure, and dynamic active-site behavior. X-ray diffraction (XRD) is commonly employed as the initial diagnostic tool to verify the amorphous nature of HEACs. Unlike crystalline materials that exhibit sharp Bragg peaks, amorphous catalysts typically lack sharp diffraction peaks or instead display broad humps at characteristic positions [65]. To further confirm the amorphous structure and gain nanoscale insights, transmission electron microscopy (TEM) serves as an essential technique. In high-resolution TEM (HRTEM), amorphous phases lack lattice fringes, and their selected area electron diffraction (SAED) patterns exhibit diffuse halo rings, unlike the sharp rings of crystalline materials. Fast Fourier transform (FFT) analysis further distinguishes the two, with amorphous regions showing ring-like features. Additional techniques such as EDS mapping and STEM (HAADF/BF) imaging provide complementary insights into the compositional uniformity and non-periodic contrast characteristic of amorphous domains. For example, EDS mapping first confirmed the presence and uniform distribution of all constituent metals, indicating the successful formation of the high-entropy metallic glass (Fig. 3a) [66]. The amorphous structure was further supported by the SAED pattern, which exhibited diffuse halos instead of distinct diffraction spots (Fig. 3b). This was corroborated by the HRTEM imaging and the corresponding FFT pattern (Fig. 3c), both of which revealed the absence of long-range order.

X-ray absorption spectroscopy (XAS), encompassing both X-ray absorption near-edge structure (XANES) and extended X-ray absorption fine structure (EXAFS), provides valuable insights into the oxidation states and coordination geometries of individual metal species, enabling detailed analysis of the complex multimetallic landscape in HEACs [67]. Surface chemical states and electronic interactions among constituent elements are further elucidated by XPS, which reveals surface redox properties and the electronic modulation induced by multimetal synergy. Electron paramagnetic resonance (EPR) spectroscopy is utilized to detect and quantify structural defects such as oxygen vacancies and unpaired electrons. In parallel, vibrational spectroscopies such as Raman and Fourier transform infrared (FTIR) spectroscopy offer complementary information by identifying functional groups and local bonding environments. Finally, to bridge static characterization with real-time catalytic behavior, in-situ and operando techniques—such as in-situ FTIR, in-situ Raman, and in-situ XRD—are essential [68]. These dynamic tools allow for the direct observation of structural evolution, active-site transformation, and intermediate formation under realistic electrochemical conditions, providing vital evidence to link structure with function.

## Synthetic Strategies of High-Entropy Amorphous Catalysts

The rational synthesis of HEACs is critical to harnessing their structural complexity and catalytic potential. Unlike crystalline materials, amorphous systems require non-equilibrium or kinetically controlled conditions to prevent long-range ordering, while the incorporation of multiple principal elements demands strategies that ensure homogeneous distribution and phase stability. To this end, diverse synthetic routes—including electrodeposition, hydrothermal/solvothermal methods, ball milling, melt spinning with dealloying, and chemical reduction—have been developed to tailor the atomic disorder, composition, and morphology of HEACs. This section reviews representative techniques for constructing HEACs, summarizing their key features, amorphization mechanisms, advantages, and limitations (Table 1).

**Table 1:** Comparison of the five principal synthetic strategies used for high-entropy amorphous catalysts

Synthetic strategies	Key features	Amorphization mechanisms	Advantages	Limitations
Electrodeposition	Controllable film thickness, direct growth on substrates	Control reduction potential and reaction time	Mild, scalable, convenient operation	Reliance on conductive substrates, potential composition non-uniformity
Hydrothermal/solvothermal methods	High temperature with morphology control	Control temperature and reaction time	Versatile morphology-controllable	Relatively high-energy input, inherent safety risk
Melt spinning with dealloying	Rapid solidification and selective dealloying	High cooling rate above 10^6^ K/s	Stable process Scalable, precise amorphous control	Complex process, high cost, limited to alloy processing
Ball milling	Solvent-assisted mechanochemistry for atomic-level mixing	High-energy mechanical collisions	Simple scalable flexible precursor	Contamination risk, High-energy consumption, uneven amorphization
Chemical reduction	Solution-phase synthesis using strong reductants	Fast reduction kinetics	Mild, high tunability, good composition control	Reducing agent contamination, poor reproducibility

### Electrodeposition

**Fig. 4:**
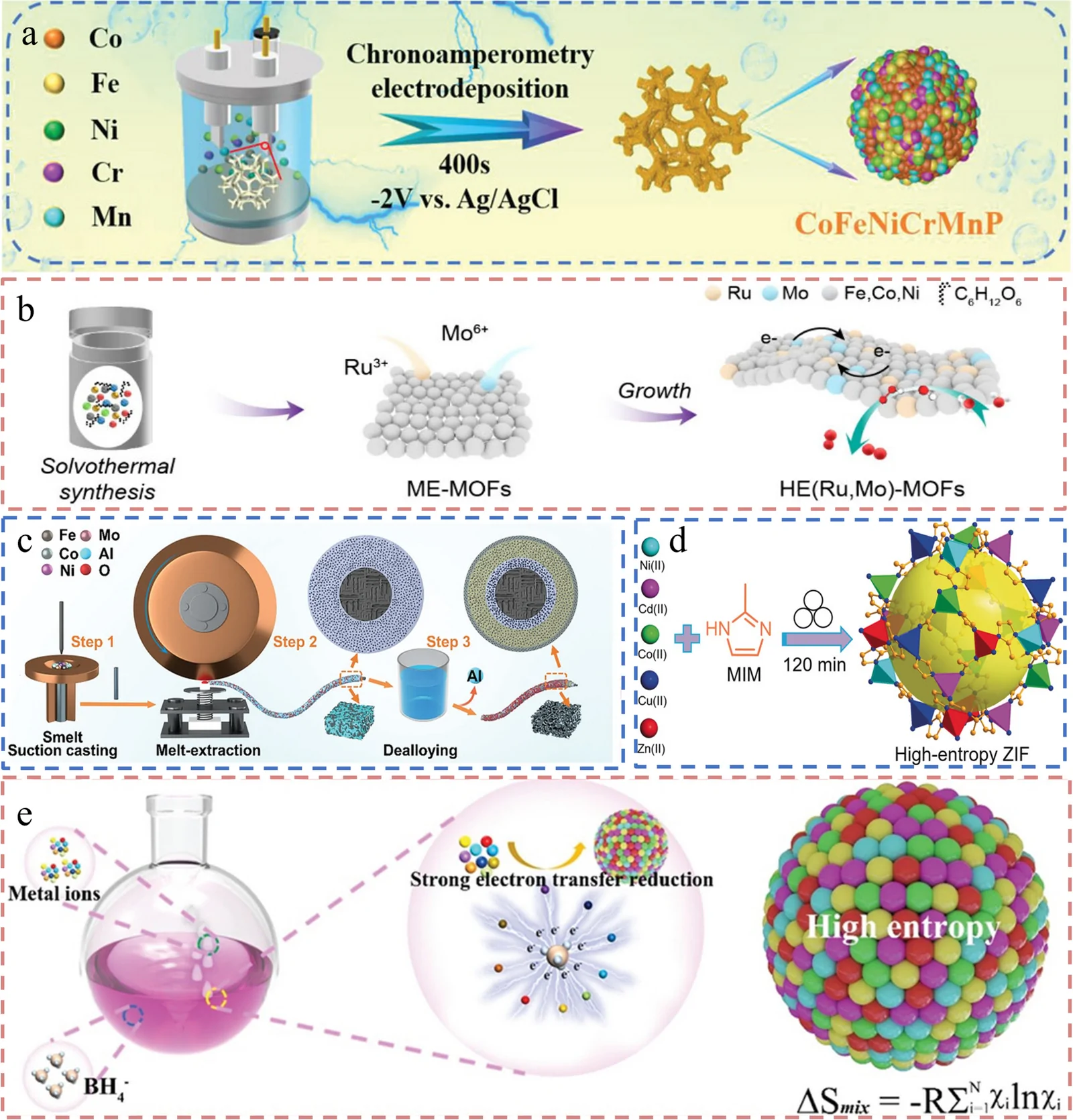
Schematic diagram of representative synthetic strategies of HEACs. **a** Electrodeposition. Reproduced with permission [74]. Copyright 2023, John Wiley and Sons. **b** Hydrothermal/solvothermal method. Reproduced with permission [83]. Copyright 2024, American Chemical Society. **c** Melt spinning with dealloying. Reproduced with permission [57]. Copyright 2023, John Wiley and Sons. **d** Ball milling. Reproduced with permission [95]. Copyright 2024, John Wiley and Sons. **e** Chemical reduction. Reproduced with permission [99]. Copyright 2024, John Wiley and Sons

Electrodeposition is a mild and scalable technique that enables the direct reduction of metal precursors onto conductive substrates through convenient operation [69, 70]. Owing to the differences in reduction potentials, reaction time, and ion diffusion during deposition, this method inherently favors the formation of amorphous phases by disrupting long-range atomic order [71, 72]. Electrodeposition technique is particularly suitable for the fabrication of self-supporting catalysts, as it enables the direct growth of uniform and firmly adhered catalytic films on conductive substrates. For instance, a high-entropy amorphous FeCoCrNi thin-film catalyst synthesized via electrodeposition exhibited uniform elemental distribution and notable electrocatalytic activity [73]. This strategy is also effective for fabricating phosphorus-modified amorphous compounds, such as phosphides and phosphoxides, offering a low-temperature, solution-based route to high-performance electrocatalysts. Li et al. developed a phosphorus-modified amorphous high-entropy CoFeNiCrMn compound through a one-step electrodeposition strategy (Fig. 4a), which exhibited superior performance in hydrazine-assisted water electrolysis [74]. Similarly, Zhang et al. employed a facile electrochemical deposition approach to prepare amorphous high-entropy phosphoxides supported on nickel foam (CNFMPO), comprising Co, Ni, Fe, Mn, P, and O [75]. The electrodeposition time was optimized to 13 min, as shorter (8 min) and longer (25 min) durations led to denser nano-lamellar structures with fewer active sites and lower mass transfer efficiency. In multicomponent systems, electrodeposition often encounters challenges in achieving precise compositional control, as variations in the reduction potentials of different metal ions lead to inconsistent deposition rates and potential compositional segregation. Moreover, the technique is inherently limited to conductive substrates, requiring additional conductive treatments for insulating materials, which constrains its broader applicability.

### Hydrothermal/Solvothermal Methods

The hydrothermal/solvothermal methods are effective and versatile approaches for synthesizing amorphous high-entropy compounds, offering excellent adaptability to various compositions and solvents [76, 77]. By conducting chemical reactions in sealed autoclaves at elevated temperatures (120–160 °C) and pressures—using water (hydrothermal) or organic solvents (solvothermal) as reaction media—this method facilitates the controlled nucleation and growth of multicomponent materials with homogeneous elemental distribution [78–80]. By adjusting parameters such as the reaction temperature and time, solvent composition, functional additive, and regulator, the morphology of resulting materials can be precisely tailored. For instance, an amorphous nanoflower-structured high-entropy material, FeCoNiMnRu-TA, was synthesized via a hydrothermal method using trimesic acid (TA) as the ligand and cetyltrimethylammonium chloride (CTAC) as the growth regulator [53]. The functionalization with the TA ligand accelerated the catalytic kinetics, enhanced structural and environmental stability, and suppressed the dissolution of metal ions. Meanwhile, the introduction of CTAC effectively directed the formation of the nanoflower morphology, resulting in improved particle dispersion. Additionally, both Ting et al. and Nguyen et al. employed the solvothermal method to synthesize high-entropy metal glycerates, which served as high-performance electrocatalysts for the oxygen evolution reaction (OER) [81, 82]. A high-entropy metal–organic framework (HE(Ru,Mo)-MOF), featuring atomically dispersed Ru and Mo sites anchored within amorphous high-entropy MOF nanosheets, was also successfully synthesized via a simple hydrothermal method (Fig. 4b) [83]. The hydrothermal/solvothermal methods typically require prolonged reactions under high-temperature and high-pressure conditions, which not only reduce synthesis efficiency but also increase energy consumption. Furthermore, the limited volume of autoclaves constrains large-scale production and poses inherent safety risks, thereby restricting its industrial applicability.

### Melt Spinning with Dealloying

Melt spinning combined with dealloying is an effective two-step strategy for synthesizing amorphous high-entropy compounds with hierarchical porous architectures [84, 85]. In this approach, a precursor alloy is first rapidly solidified via melt spinning, which utilizes high cooling rates (typically above 10^6^ K s^−1^) to suppress atomic diffusion and crystallization, resulting in the formation of a metastable amorphous ribbon [86, 87]. Subsequently, selective chemical or electrochemical dealloying is applied to remove specific elements from the alloy matrix, introducing nanoscale porosity while preserving the amorphous framework. This process not only enhances surface area and mass transport but also exposes a high density of catalytically active sites. For example, Cui et al. utilized high-purity Fe, Co, Ni, Mo, and Al (4N) to prepare precursor ingots via arc melting (Fig. 4c), followed by the fabrication of metallic fibers through melt extraction under deep undercooling [57]. A porous surface with core–shell structures was subsequently introduced by dealloying, enhancing the specific surface area and exposing more catalytic active sites. Yu et al. also employed a melt-spinning followed by dealloying strategy to synthesize amorphous-crystalline high-entropy alloys (AC-HEAs) composed of Cu, Ag, Au, Pt, Pd, Ir, and Ru [88]. During dealloying, selective leaching of active Y and Al atoms produced water-soluble ions, while the released noble metal atoms diffused along the reaction interface, leading to atomic-level reconstruction of multicomponent alloy clusters. The position of the diffraction peak in the resulting HEAs was found to depend on the average atomic radius of the constituent elements, indicative of an amorphous-like continuous solid solution phase. Although melt spinning is characterized by its stable and reproducible processing, suitability for large-scale continuous production, and ability to precisely control amorphous structure formation through ultra-high cooling rates, its application is limited to materials that can form a fluid melt with a moderate melting point, and the process typically yields only thin ribbons or fibers. Even though subsequent dealloying processes such as corrosion, oxidation, and heat treatment can be applied to the alloy to obtain porous, composite, or oxide structures, these steps increase the overall cost. Moreover, excessively thin ribbons are not suitable for post-treatment, which limits this method primarily to alloy processing.

### Ball Milling

Ball milling has been widely employed as a powerful technique for the synthesis of amorphous high-entropy compounds due to its simplicity and scalability [89, 90]. In this method, multiple metal precursors are subjected to high-energy mechanical collisions within a milling chamber, often in the presence of a suitable solvent. The solvent plays a crucial role in controlling particle agglomeration, enhancing heat dissipation, and facilitating uniform mixing of components during milling [91, 92]. The repeated fracturing, cold welding, and interdiffusion induced by the milling process disrupt the long-range crystalline order, leading to the formation of amorphous or metastable structures [93, 94]. For instance, a ball-milling approach at ambient temperature was employed to successfully synthesize a class of high-entropy zeolitic imidazolate frameworks (HE-ZIFs) (Fig. 4d), in which five different metal ions (Zn^2+^, Co^2+^, Cd^2+^, Ni^2+^, and Cu^2+^) were homogeneously incorporated into the ZIF lattice through coordination with 2-methylimidazole ligands [95]. Unlike solvothermal synthesis, which only yielded phase-separated ZIF-8/ZIF-67 composites, the mechanochemical route enabled true multicomponent mixing at the atomic level. This method leverages mechanical energy input and localized frictional heating during the ball-milling process to activate reactants and facilitate rapid diffusion, thereby promoting the formation of disordered, multimetallic frameworks without the need for prolonged high-temperature treatment. Ball milling can overcome the limitations of conventional fabrication methods, enabling high solid solubility and uniform mixing of diverse elements at low temperatures, with broad flexibility in material selection. However, the main drawbacks of high-energy ball milling include impurity contamination from the milling media or vessel during prolonged operation, long processing times and high-energy consumption to achieve adequate mixing or amorphization, and non-uniform stress distribution causing uneven amorphization.

### Chemical Reduction

Chemical reduction is a solution-based synthesis strategy that has proven particularly effective for fabricating HEACs, especially those composed of multiple transition metals [96, 97]. In contrast to solid-state or mechanochemical approaches, chemical reduction operates under milder conditions, relying on the use of reducing agents such as sodium borohydride (NaBH_4_) or hydrazine to convert metal ions into zero-valent atoms in a liquid-phase environment [98]. This process facilitates controlled nucleation and growth, while simultaneously suppressing crystallization due to rapid reaction kinetics and strong reductive driving forces. Moreover, the process is highly tunable, with parameters such as the reducing agent, solvent type, pH, and the presence of ligands or surfactants (e.g., PVP) precisely adjustable to control the morphology, particle size, and composition of the final materials. For instance, a room-temperature chemical reduction method in air using NaBH_4_, PVP, and diethylene glycol (DEG) enabled the successful synthesis of a high-entropy amorphous metal oxide [99]. During the reaction, excess NaBH_4_ overcame the reduction potential differences among various metal ions, leading to their simultaneous reduction to isolated atoms. These atoms then aggregated into nuclei and evolved into a homogeneous, brown-black amorphous oxide, as indicated by the progressive color change of the solution. Similarly, Jiang et al. extended this approach to prepare high-entropy amorphous oxide nanoparticles (HEAO-NPs) (Fig. 4e), demonstrating compositional tunability from ternary to decenary (three to ten components) systems [100]. The chemical reduction method, while advantageous for its mild conditions, rapid reaction rates, and ability to produce nanomaterials uniformly in solutions, also presents several challenges. First, rapid kinetics often lead to broad particle size distributions and irregular morphologies, making precise structural control difficult. Second, residual reducing agents and by-products can remain on the product surface, introducing impurities that compromise catalytic activity and stability. Third, the process is highly sensitive to reaction parameters such as pH, temperature, and reductant concentration, resulting in poor reproducibility. Therefore, while simple at the laboratory scale, maintaining particle uniformity and compositional consistency becomes challenging during industrial-scale production.

### Other Methods

In addition to the commonly used methods mentioned above, other strategies have also been employed to synthesize high-entropy amorphous catalysts. For example, a kinetically controlled flash carbothermic method was developed to synthesize metallic glass nanoparticles within milliseconds using ultrafast heating and cooling rates. Nine compositions (M_1_–M_2_–P, M_1_ = Pt/Pd; M_2_ = Cu/Ni/Fe/Co/Sn) were obtained with tunable sizes [66]. Compared to bulk counterparts, the nanoscale form showed enhanced glass-forming ability, highlighting the size-dependent advantage. Johny et al. employed pulsed laser ablation of bulk high-entropy alloy targets in acetonitrile to synthesize colloidal carbon-coated CrCoFeNiMn and CrCoFeNiMnMo high-entropy metallic glass nanoparticles (HEMG-NPs) [101]. These amorphous, multielemental NPs outperformed their crystalline counterparts in electrocatalytic energy conversion, demonstrating both enhanced activity and structural suitability for the water-splitting application. Similarly, high-entropy metallic glass nanoparticles (HEMG-NPs) with the composition FeCoNiCrMo_1.0_ were synthesized using a laser-evaporated inert-gas condensation (LE-IGC) technique [62]. In this process, a focused picosecond pulsed laser induced the evaporation of metal atoms from a multicomponent target. These atoms subsequently underwent initial collisions, leading to the formation of atomic clusters. The resulting small liquid metal droplets rapidly condensed into HEMG-NPs. Frequent collisions between the FeCoNiCrMo vapor and inert-gas atoms facilitated ultrafast cooling, effectively suppressing particle growth and promoting the formation of nanoscale amorphous structures.

## Reaction Mechanisms in Water Electrolysis

**Fig. 5:**
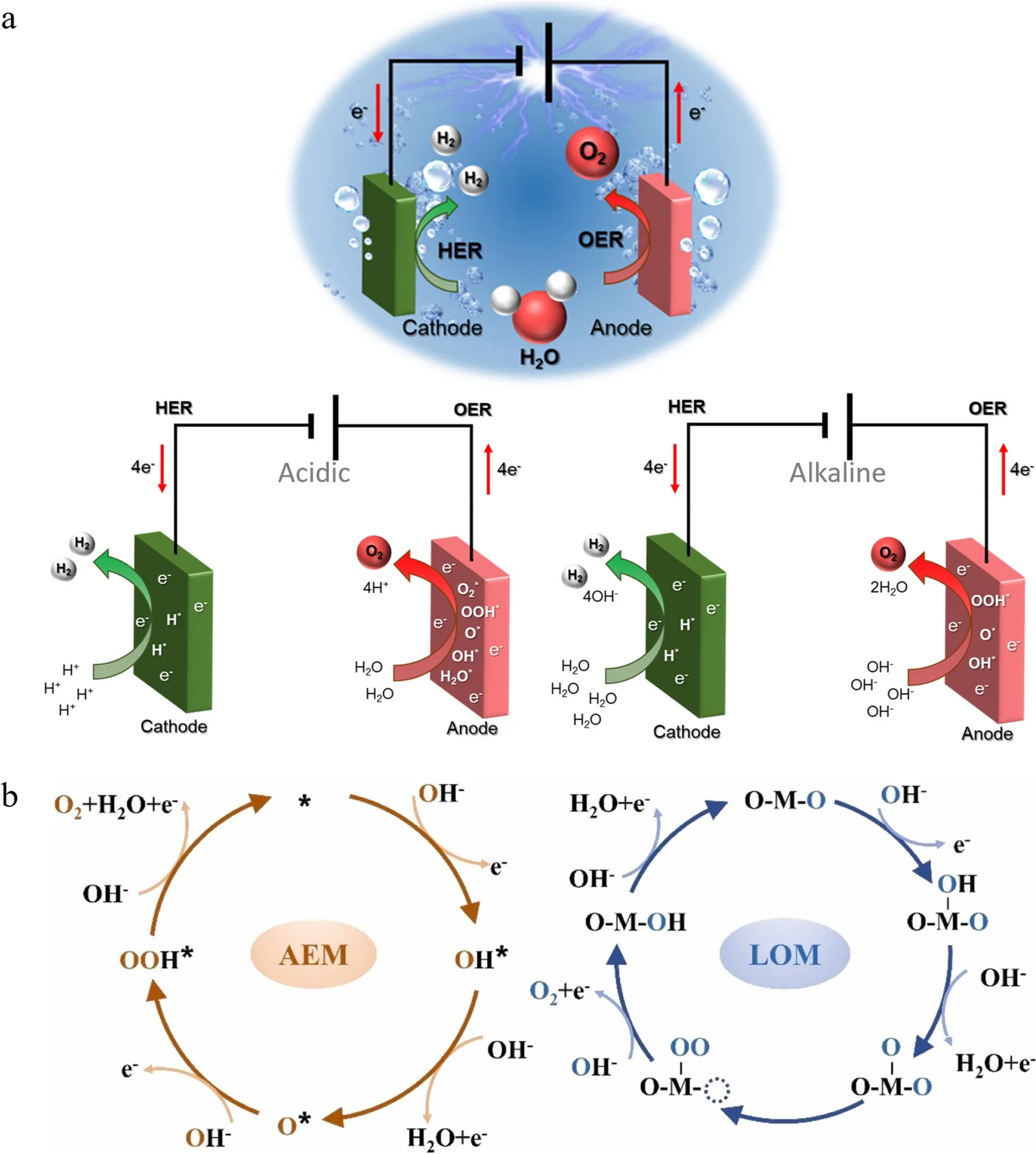
Water electrolysis mechanisms. **a** Schematic illustration of electrocatalytic water splitting consisting of HER and OER half-cell reactions [110]. **b** Schematic illustration of the AEM and LOM mechanism for the OER (alkaline conditions) [109]

Water electrolysis is a fundamental process for hydrogen production and involves two half-reactions: HER at the cathode and OER at the anode (Fig. 5a). Both reactions involve multiple proton-coupled electron transfer steps and suffer from sluggish kinetics, especially OER, which involves complex four-electron transfer pathways.

The HER proceeds via either the Volmer-Heyrovsky or Volmer-Tafel mechanisms [102–104]:

Volmer step:

3 $${\mathrm{H}}^{ + } + {\mathrm{e}}^{ - } \to {^*}{\mathrm{H}}\;\left( {\text{acidic media}} \right)$$

4 $${\mathrm{H}}_{{2}} {\mathrm{O}} + {\mathrm{e}}^{ - } \to {^*}{\mathrm{H}} + {\mathrm{OH}}^{ - } \;\left( {{\mathrm{alkaline}}\;{\mathrm{media}}} \right)$$

Heyrovsky step:

5 $${\mathrm{H}}^{ + } + {^*}{\text{H }} + {\mathrm{e}}^{ - } \to {\mathrm{H}}_{{2}} \;\left( {{\mathrm{acidic}}\;{\mathrm{media}}} \right)$$

6 $${^*}{\mathrm{H}} + {\mathrm{H}}_{{2}} {\mathrm{O}} + {\mathrm{e}}^{ - } \to {\mathrm{H}}_{{2}} + {\mathrm{OH}}^{ - } \;\left( {{\mathrm{alkaline}}\;{\mathrm{media}}} \right)$$

Tafel step:

7 $${^*}{\mathrm{H}} + {^*}{\mathrm{H}} \to {\mathrm{H}}_{{2}}$$

The HER exhibits notable differences under acidic and alkaline conditions. In acidic media, the reaction benefits from abundant protons (H⁺), enabling fast proton-coupled electron transfer via the Volmer-Tafel or Volmer-Heyrovsky mechanisms [3, 105]. As a result, HER kinetics are generally more favorable, and overpotentials are lower. In contrast, under alkaline conditions, water molecules act as the proton source, and an additional water dissociation step is required to generate adsorbed hydrogen intermediates. This step introduces a kinetic bottleneck, making HER inherently slower in alkaline media.

The OER in alkaline conditions generally follows this reaction pathway [106]:

8 $${\mathrm{OH}}^{ - } \to {^*}{\text{OH }} + {\mathrm{e}}^{ - }$$

9 $${^*}{\mathrm{OH}} + {\mathrm{OH}}^{ - } \to {^*}{\mathrm{O}} + {\mathrm{H}}_{{2}} {\mathrm{O}} + {\mathrm{e}}^{ - }$$

10 $${^*}{\mathrm{O}} + {\mathrm{OH}}^{ - } \to {^*}{\mathrm{OOH}} + {\mathrm{e}}^{ - }$$

11 $${^*}{\mathrm{OOH}} + {\mathrm{OH}}^{ - } \to {\mathrm{O}}_{{2}} + {\text{ e}}^{ - } + {\mathrm{H}}_{{2}} {\mathrm{O}}$$

In acidic environments, the OER mechanisms differ slightly due to the higher proton concentration [107]:

12 $${^*}{\mathrm{H}}_{{2}} {\text{O }} \to {\text{ H}}^{ + } + \, {^*}{\text{OH }} + {\text{ e}}^{ - }$$

13 $${^*}{\mathrm{OH}} \to {^*}{\mathrm{O}} + {\mathrm{H}}^{ + } + {\mathrm{e}}^{ - }$$

14 $${^*}{\mathrm{O}} + {\mathrm{H}}_{{2}} {\mathrm{O}} \to {^*}{\mathrm{OOH}} + {\mathrm{e}}^{ - } + {\text{ H}}^{ + }$$

15 $${^*}{\text{OOH }} \to {\text{ O}}_{{2}} {^*} + {\text{ e}}^{ - } + {\text{ H}}^{ + }$$

16 $${\mathrm{O}}_{{2}} {^*} \to {^*} + {\mathrm{O}}_{{2}}$$

The OER also shows distinct behavior under acidic and alkaline conditions. The higher conductivity and lower resistance of acidic electrolytes often lead to more favorably charged transport. However, the aggressive environment poses challenges for long-term system stability. In contrast, alkaline conditions offer a milder chemical environment but involve different mechanistic features. Here, hydroxide ions serve as the oxygen source, and the OER proceeds via the adsorption and deprotonation of OH⁻ [108]. Although the alkaline environment broadens the operational window and allows for more stable reactions, the multi-step deprotonation and electron transfer processes can result in slower kinetics compared to acidic systems. Furthermore, the pH-dependent formation energy and binding strength of reaction intermediates may influence the rate-determining step, leading to distinct mechanistic pathways under each condition.

The OER generally proceeds via two fundamental mechanisms: the adsorbate evolution mechanism (AEM) and the lattice oxygen mechanism (LOM) (Fig. 5b) [109]. Both routes involve the stepwise generation and transformation of surface‐bound catalytic oxygen species (COSs) leading to the formation of molecular oxygen. Using alkaline conditions as an example, the AEM pathway typically initiates from a vacant metal active site (*), where hydroxide ions (OH⁻) from the electrolyte are adsorbed, forming an M–OH intermediate through a single electron transfer process. This intermediate subsequently undergoes deprotonation and electron transfer via a nucleophilic attack by OH⁻, producing the M–O* species. Continued reaction with another OH⁻ leads to O–O bond formation, resulting in an M–OOH* intermediate. Final dehydrogenation of this intermediate releases O_2_ and regenerates the active site, thus completing the catalytic cycle. In certain cases, especially for catalysts with surfaces rich in hydroxide or hydrous oxide species, these pre-existing hydroxyl groups can act as initial active modes for OER. In such situations, M–OH* may serve as the starting point of the catalytic turnover, though this variation does not significantly change the COS formation pathway. However, it may influence the interpretation of electrokinetic behavior. Notably, the structural identity of surface hydroxide species affects whether a reaction is categorized under AEM or LOM. If the hydroxide is terminally bound to a single-metal site, its evolution typically falls under AEM. In contrast, if the hydroxyl bridges between two metal centers and evolves into a bridging oxygen species, the reaction is more appropriately described by the LOM pathway.

## Applications of High-Entropy Amorphous Catalysts in Water Splitting

We present a table that provides a clear and intuitive overview of the catalytic behaviors of various high-entropy amorphous catalysts (Table 2). To gain deeper insights into these structure–activity relationships, the following sections classify the catalysts into six categories based on their modifications and architectures: (1) oxides/hydroxides, (2) non-oxide anions such as P, B, and F, (3) oxyanions including PO_4_^3−^, glycerate, BO_3_^3−^, and CO_3_^2−^, (4) MOF-based modifications, and (5) heterostructured composites. Representative examples from each category are discussed in detail to highlight their structural characteristics, catalytic performances, and underlying mechanisms.

**Table 2:** Overview of the catalytic behaviors of various high-entropy amorphous catalysts

HECs	Synthesis techniques	Overpotential [mV]	Electrolyte	Tafel slope [mV dec^−1^]	Reaction	References
FeCoNiMnBOx	Chemical reduction	*η*_10_ = 266	1 M KOH	64.5	OER	[100]
NiFeCoMnAl oxide	Electrodeposition and dealloying	*η*_10_ = 190	1 M KOH	47.62	OER	[111] [53]
		*η*_10_ = 390	0.5 M KHCO_3_	126.61	OER	
FeCoNiMnRu-TA	Hydrothermal method	*η*_100_ = 226	1 M KOH	24.4	OER	[53]
IrRuCrFeCoNiOx	Coprecipitation	*η*_10_ = 190	1 M KOH	51.1	OER	[112]
CrCoFeNiMnMo	Laser ablation	*η*_6_ = 470	0.1 M NaOH		OER	[101]
FeCoNiPB oxide	Chemical reduction	*η*_10_ = 235	1 M KOH	53	OER	[40]
NiFeCoMoAl	Dealloying	*η*_2000_ = 470	1 M KOH	39.8	OER	[57]
AC-HEA-CuAgAuIrRu	Dealloying	*η*_10_ = 9.5	0.5 M H_2_SO_4_	32	HER	[88]
		*η*_10_ = 20	1 M KOH	33	HER	
		*η*_10_ = 208	0.5 M H_2_SO_4_	40	OER	
		*η*_10_ = 200	1 M KOH	57	OER	
FeCoNiMnCu-82	Chemical reduction	*η*_10_ = 259	1 M KOH	74.8	OER	[99]
FeCoNiMoVOx-1.5	Hydrothermal method	*η*_10_ = 216	1 M KOH	54.49	OER	[60]
		*η*_10_ = 231	1 MKOH + 0.5 M NaCl	–	OER	
		*η*_10_ = 236	1 M KOH + seawater	–	OER	
AlNiCuCoFeYFx	Melt spinning with dealloying	*η*_10_ = 261	1 M KOH	50	OER	[113]
HEO-110	Hydrothermal method	*η*_10_ = 290	1 M KOH	85	OER	[114]
PdPtCuNiP	B_2_O_3_ flux	*η*_10_ = 35.4	0.5 M H_2_SO_4_	34.2	HER	[115]
CoFeNiCrMnP/NF	Electrodeposition	*η*_100_ = 51	1 M KOH	48	HER	[74]
FeCoNiMnBPOx	Chemical reduction	*η*_10_ = 248	1 M KOH	42.3	OER	[54]
FeCoNiCrVB	Arc melting and roller spinning	*η*_10_ = 237	1 M KOH	24.2	OER	[116]
FeCoCrMnBS	Electrodeposition and chemical reduction	*η*_100_ = 290	1 M KOH	51.4	OER	[117]
HE-CNO	Coprecipitation and calcination	*η*_10_ = 328	1 M KOH	70	OER	[118]
(FeNiCuCoZn)_90-x_V_x_P_10_	Ball milling	*η*_10_ = 228	1 M KOH	23.6	OER	[119]
FeCoCrNi thin films	Electrodeposition	*η*_10_ = 295	1 M KOH	69.52	OER	[73]
HE(Ru,Mo)-MOFs	Hydrothermal method	*η*_10_ = 267	1 M KOH	–	OER	[83]
		*η*_10_ = 266	1.0 M KOH wastewater	–	OER	
CoCrFeMnMoCO_3_	Hydrothermal method	*η*_10_ = 302	1 M KOH	36.7	OER	[120]
Fe_29_Co_27_Ni_23_Si_9_B_12_	Melt spinning	*η*_10_ = 230	1 M KOH	85	OER	[121]
HEA/Ni_2_P	Hydrothermal method	*η*_10_ = 255	1 M KOH	56.86	OER	[122]
FeNiCoCrMnS_2_	Solvothermal method	*η*_10_ = 199	1 M KOH	39.1	OER	[123]
FeNiCoCrMn-G	Hydrothermal method	*η*_100_ = 278	1 M KOH	40	OER	[82]
CrMnFeCoNi HEGs	Solvothermal method	*η*_50_ = 251	1 M KOH	42.3	OER	[81]
CoFeNiMnMoPi	High temperature fly-through method	*η*_10_ = 270	1 M KOH	74	OER	[124]
CoCrFeNiMo	Hydrothermal method	*η*_10_ = 220	1 M KOH	30.3	OER	[125]
PtRhPdIrRu	Chemical reduction	*η*_10_ = 25	0.5 M H_2_SO_4_	22.6	HER	[126]
Fe_38_Ni_5_Co_31_Mo_15_W_11_	Eelectrodeposition	*η*_10_ = 243	1 M KOH	83	HER	[127]
		*η*_10_ = 171	1 M KOH	110	HER	
		*η*_10_ = 188	1 M KOH	106	HER	
FeCoNiCuMnPx/C	Solvothermal method and calcination	*η*_10_ = 239	1 M KOH	109.6	OER	[128]
HEBOx/MXene	Etching and chemical reduction	*η*_10_ = 268	1 M KOH	39.8	OER	[129]
HEO-Zr_1.0_	Coprecipitation	*η*_10_ = 257	1 M KOH	40.3	OER	[130]
		*η*_10_ = 181	1 M KOH	–	HER	

### High-entropy Oxides/Hydroxides

**Fig. 6:**
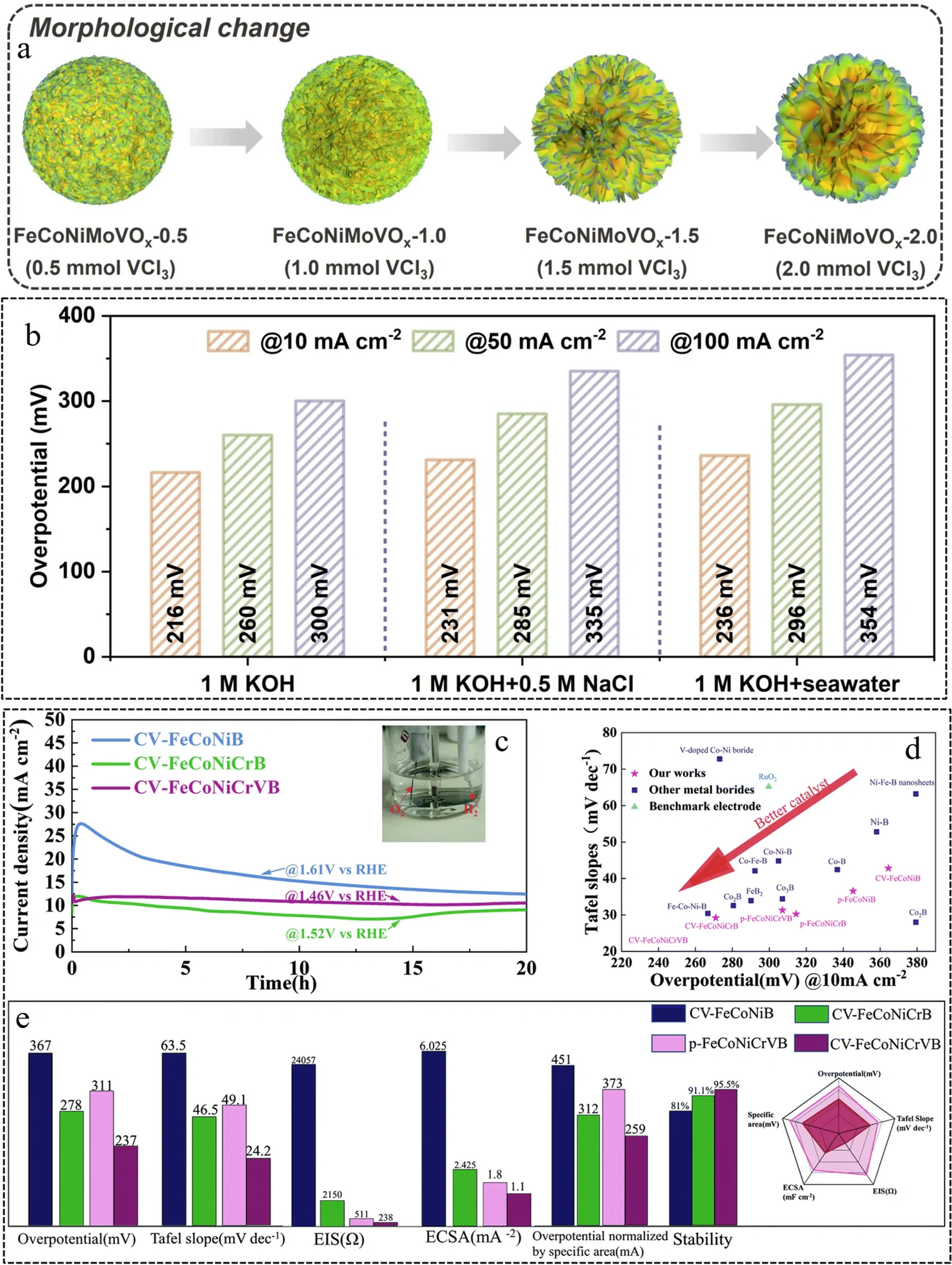
Catalytic performance evaluation. **a** Morphological change resulted from different V amount. **b** OER overpotentials measured at current densities of 10, 50, and 100 mA cm^−2^ across various electrolytes. Reproduced with permission [60]. Copyright 2024, Elsevier. **c** Chronoamperometric profiles of CV-FeCoNiB, CV-FeCoNiCrB and CV-FeCoNiCrVB. **d** Comparison of overpotentials at 10 mA cm^−2^ and Tafel slopes between this study and other recently reported OER catalysts. **e** The summary histogram of catalyst performance. Reproduced with permission [116]. Copyright 2022, Royal Society of Chemistry

High-entropy oxides (HEOs) and hydroxides (HEHs) are among the most extensively studied classes of high-entropy amorphous catalysts for water splitting, particularly due to their structural versatility, redox activity, and electrochemical stability. Their amorphous forms offer numerous active sites, disordered coordination environments, and flexible oxygen bonding networks that enhance catalytic performance in both HER and OER. The elemental ratio in high-entropy metal oxides plays a pivotal role in determining their structural morphology, electronic structure, and catalytic activity. Fine-tuning the composition can modulate the oxidation states of active metals and influence the formation of catalytically favorable architectures, thereby enhancing OER performance. For instance, a series of FeCoNiMoVO_x_/NF composites were synthesized to investigate the role of vanadium incorporation (Fig. 6a). Progressive addition of V not only transformed the catalyst morphology from microspheres to 3D microflowers but also increased the proportion of high-valent Fe, Co, and Ni species, which are critical for active oxy(hydroxide) intermediate formation [60]. The optimized sample, FeCoNiMoVO_x_-1.5, exhibited a well-defined microflower structure with enlarged inter-nanosheet voids, facilitating mass transport and gas release. It achieved low overpotentials of 216 and 236 mV at 10 mA cm^−2^ in alkaline water and natural seawater, respectively, and sustained 1 A cm^−2^ OER performance over 100 h in seawater, demonstrating excellent activity and durability (Fig. 6b). Notably, excessive V incorporation (FeCoNiMoVO_x_-2.0) led to structural aggregation and a decline in the high-valence metal ratio, underscoring the importance of compositional optimization.

During the OER, amorphous high-entropy oxides frequently undergo surface reconstruction, leading to substantial changes in their structural and catalytic properties. This reconstruction is often driven by the selective leaching or oxidation of specific metal or non-metal elements, which initiates a transformation of the surface layer into metal (oxy)hydroxide phases under electrochemical conditions. For instance, Zhang et al. synthesized an amorphous FeCoNiMoW high-entropy oxide (FCNMWO) catalyst and observed that Mo leaching during OER operation triggered surface reconstruction. The formation of MOOH species was identified as the true catalytically active phase [131]. Similarly, Li et al. demonstrated that the dissolution of boron and phosphorus accelerated surface self-reconstruction in FeCoNiMnBPO_x_ [54]. The resulting generation of high-valence metal-OOH intermediates contributed to improved catalytic activity. Furthermore, Rong et al. confirmed that surface reconstruction during OER led to the formation of a crystalline active layer several nanometers thick, while the underlying amorphous core remained intact, offering a balance between activity and durability [112].

### High-entropy Anionic Non-oxides

Recent advances have highlighted the strategic potential of introducing non-oxygen anions—such as phosphide (P), sulfide (S), and boride (B)—into high-entropy systems to enhance their electrocatalytic performance for water splitting [37, 132, 133]. These anions, due to their lower electronegativity and more flexible coordination geometries compared to oxygen anions, enable refined modulation of the local electronic environment [134, 135]. A representative example is the synthesis of a sulfate-modified high-entropy sulfide, FeNiCoCrMnS_2_, via a two-step solvothermal method using earth-abundant metals [123]. This material delivered superior OER performance, achieving low overpotentials ranging from 199 to 308 mV across a broad current density window (10–1000 mA cm^−2^), significantly outperforming its unary to quaternary metal sulfide counterparts. Remarkably, the catalyst retained excellent stability over 12,000 cyclic voltammetry cycles and sustained operation at 500 mA cm^−2^ for 55 h. In-situ and ex-situ characterizations revealed that the high activity was associated with surface reconstruction into active metal (oxy)hydroxides, while residual sulfate species contributed synergistically to the catalytic enhancement.

**Fig. 7:**
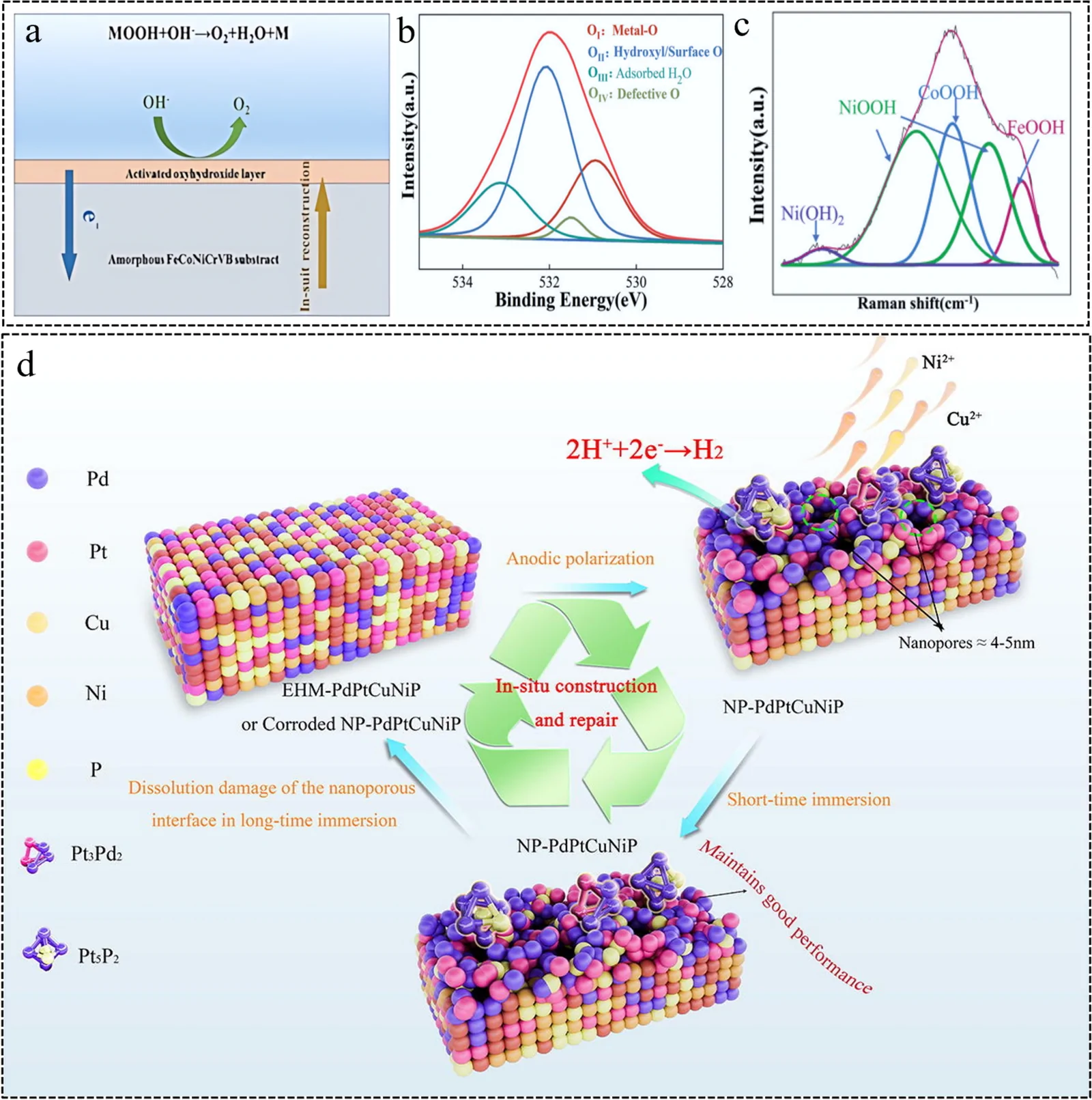
Reconstruction of HEACs. **a** Schematic description of the reconstruction process of p-FeCoNiCrVB. **b** XPS spectra of O1s (O_I_: metal oxides, O_II_: hydroxyl/surface oxygen, O_III_: adsorbed oxygen and O_IV_: defect oxygen). **c** Raman spectra for CV-FeCoNiCrVB. Reproduced with permission [116]. Copyright 2022, Royal Society of Chemistry. **d** Schematic representation of the in-situ formation and self-reconstruction of high-entropy amorphous alloys Reproduced with permission [115]. Copyright 2023, Elsevier

Similarly, an amorphous high-entropy FeCoNiCrVB catalyst exhibited pronounced OER enhancement through cyclic voltammetry-induced surface reconstruction [116]. Chronoamperometry tests reveal that CV-FeCoNiCrVB maintained excellent stability over 20 h without noticeable degradation, surpassing CV-FeCoNiCrB and CV-FeCoNiB (Fig. 6c). The enhanced electron transport and interfacial stability, induced by structural modifications, contribute to a low overpotential of 237 mV at 10 mA cm^−2^—outperforming both its simpler analogues and commercial RuO_2_ (Fig. 6d, e). The reconstruction process involved selective leaching of B and V, formation of high-valent metal (oxy)hydroxide species, and the development of a hierarchical core–shell structure with a sandwich-like interface (Fig. 7a) [116]. XPS analysis revealed the generation of abundant surface hydroxyl groups (O_II_, 532.1 eV) on CV-FeCoNiCrVB (Fig. 7b), which are regarded as real active sites for the OER process. This finding was further corroborated by the Raman spectra of CV-FeCoNiCrVB after 1000 CV cycles, which can be deconvoluted into five characteristic peaks assigned to NiOOH (358 cm^−1^), Ni(OH)_2_ (494 and 589 cm^−1^), CoOOH (542 cm^−1^), and FeOOH (649 cm^−1^)—all of which were well-established OER-active species (Fig. 7c) [136].

For hydrogen evolution reaction (HER), a high-entropy metallic glass composed of equiatomic PdPtCuNiP was employed to construct a nanoporous sponge-like architecture via potentiostatic electrochemical dealloying [115]. The resulting material demonstrated exceptional HER performance in hot dilute H_2_SO_4_, achieving a low overpotential of 35.4 mV at 10 mA cm^−2^ and a Tafel slope of 34.2 mV dec^−1^, exceeding the performance of commercial Pt/C. Importantly, even after mild degradation under intermittent or low-voltage operation, the catalyst’s activity could be effectively regenerated through in-situ anodic polarization, highlighting its practical viability for sustainable HER applications in corrosive environments powered by fluctuating renewable-energy inputs (Fig. 7d). During potentiostatic polarization at 1.25 V vs. RHE in deaerated 0.5 mol L^−1^ H_2_SO_4_ at 25 °C, selective corrosion evolved progressively. After 0.5 h, nanopores (3–7 nm radius, 2–6 nm depth) formed from partial dissolution of surface Cu and Ni. At 2 h, continued dissolution of Cu, Ni, and partial Pd enlarged pores to 4–12 nm wide and 4–10 nm deep. By 6 h, sustained selective dissolution drove outward migration of sub-surface Cu and Ni, creating a 3D nanoporous structure. Simultaneously, residual Pd, Pt, and P reconstructed to form Pd_3_Pt_2_ and Pt_5_P_2_ nanocrystals.

### High-entropy Metal–organic Frameworks

High-entropy metal–organic frameworks (HE-MOFs) have emerged as a promising class of electrocatalysts for the OER, owing to their unique structural versatility and multicomponent composition [137, 138]. The incorporation of multiple metal species in near-equimolar ratios gives rise to a high configurational entropy effect, which contributes to structural stabilization, suppression of phase segregation, and enhanced durability under harsh oxidative conditions. In addition, the inherently porous architecture of MOFs provides a large specific surface area and uniform dispersion of active sites, while the chemical flexibility of the framework enables tailored modification of catalytic properties [139, 140].

**Fig. 8:**
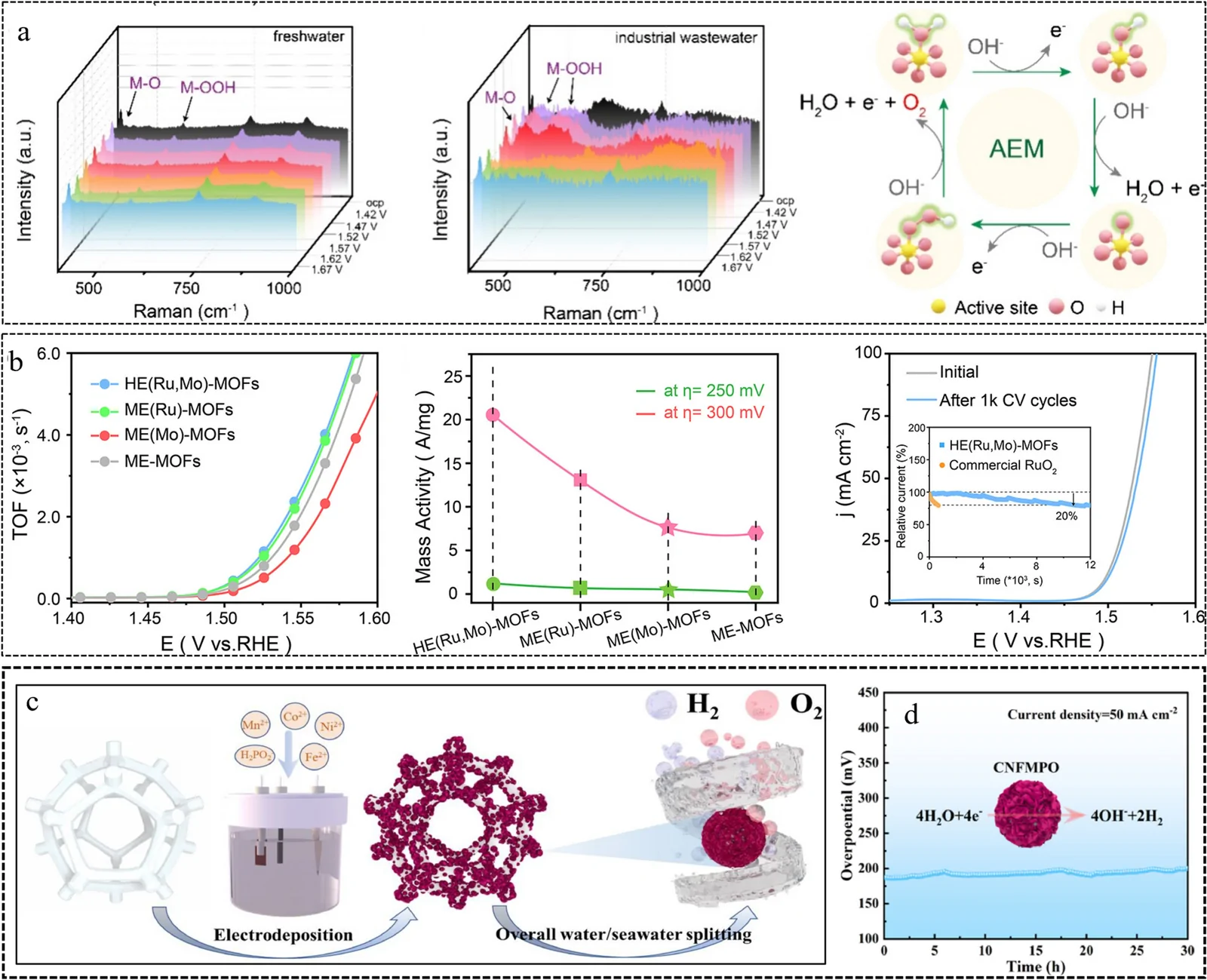
Performance evaluation of HEACs**. a** In-situ Raman spectra of HE(Ru,Mo)-MOFs in alkaline freshwater and wastewater electrolytes at different applied potentials and models of the different intermediates in the AEM pathway. **b** Comparisons of TOF and mass activity at different potentials between HE(Ru,Mo)-MOFs and control samples in 1.0 M KOH wastewater electrolyte; CV cycling and chronoamperometry (inset) of HE(Ru,Mo)-MOFs in 1.0 M KOH wastewater electrolyte. Reproduced with permission [83]. Copyright 2024, American Chemical Society. **c** Schematic illustration of the fabrication process of CNFMPO. **d** Chronopotentiometric profile of CNFMPO recorded at a current density of 50 mA cm^−2^ in 1.0 M KOH. Reproduced with permission [75]. Copyright 2024, Elsevier

Mu et al. designed a high-entropy metal–organic framework (HE(Ru,Mo)-MOF) in which atomically dispersed Ru and Mo sites were anchored within amorphous high-entropy MOF nanosheets and further stabilized by in situ-formed amorphous high-entropy oxides [83]. Comprehensive structural and electrochemical characterizations—including in-situ Raman and operando electrochemical impedance spectroscopy—revealed the presence of high-density O-bridged Ru-Mo dual-atom active sites (Fig. 8a). The multimetallic framework induced electronic redistribution, which effectively regulated the oxidation states of the active metal centers and consequently enhanced the intrinsic OER activity. Such atomic dispersion and electronic tuning collectively promoted rapid charge transfer and the dynamic transformation of oxygenated intermediates during catalysis. HE(Ru, Mo)-MOFs exhibited remarkable catalytic performance, delivering low overpotentials of 267 and 266 mV at 10 mA cm^−2^ in alkaline freshwater and industrial wastewater, respectively. These values not only surpassed those of conventional mono- and bimetallic MOFs but also outperformed commercial RuO_2_. Notably, the catalyst maintained outstanding operational stability under prolonged OER testing, demonstrating its potential for scalable and durable wastewater electrolysis applications (Fig. 8b). Although HE(Ru, Mo)-MOFs demonstrated a stable amorphous structure during long-term electrochemical testing, with no obvious aggregation or degradation of the nanosheets, and electrochemical impedance spectroscopy (EIS) measurements indicated consistently low charge-transfer resistance—confirming the catalyst's robust operational stability—the potential structural evolution of MOF under electrochemical conditions remains an important issue [141]. This aspect warrants further investigation in future studies on MOF-derived catalysts.

### High-entropy Oxyanion Compounds

High-entropy oxyanion compounds, which integrate multiple metal cations with oxygen-containing anions (such as phosphate (PO_4_^3−^), glycerate (C_3_H_5_O_4_^−^), borate (BO_3_^3−^) and carbonate (CO_3_^2−^)), represent a promising class of materials for electrochemical water splitting [142, 143]. The combination of high configurational entropy and diverse oxyanionic coordination environments enables structural stabilization, flexible electron modulation, and multi-site activation under electrochemical conditions [144, 145]. A Co/Ni/Fe/Mn-based amorphous high-entropy phosphoxide (CNFMPO) electrode was synthesized via a rapid electrodeposition method (Fig. 8c) [75]. CNFMPO demonstrated outstanding bifunctional electrocatalytic activity in alkaline water and seawater, requiring low overpotentials of 43/73 mV for HER and 252/282 mV for OER at 10 mA cm^−2^. In a two-electrode configuration, CNFMPO achieved overall water/seawater splitting at 1.54/1.56 V with excellent stability. Notably, CNFMPO demonstrated excellent durability during prolonged electrolysis (Fig. 8d), attributed to in-situ surface reconstruction into catalytically active metal (oxy)hydroxides, phosphite, and phosphates [75]. The reconstructed phosphate/phosphite species further contributed to inhibiting hypochlorite formation during seawater electrolysis, ensuring selectivity and operational safety [132]. An amorphous high-entropy metal borate, (CrMnCoNiFe)_0.2_BO_x_, was synthesized via a simple solvothermal method [146]. It possessed a high surface area (446 m^2^ g^−1^) with porous structure and exhibited excellent OER performance with a low overpotential (236 mV at 10 mA cm^−2^), a Tafel slope of 64 mV dec^−1^, and good stability. The enhanced activity was attributed to its high-entropy structure, Cr vacancy formation, and in-situ reconstruction into active metal (oxy)hydroxides, as confirmed by in-situ Raman, XPS, TEM, and EPR. DFT calculations showed that the high-entropy matrix reduced the energy barriers for oxygen intermediate transformations, in agreement with experimental findings.

Metal glycerates, a representative subclass of metal alkoxides, have emerged as versatile intermediate templates for synthesizing nanomaterials with tunable morphologies for diverse functional applications [81]. Structurally, these compounds feature a layered architecture comprising stacked metal–oxygen sheets interspersed with intercalated glycerate anions. In high-entropy metal glycerates, the incorporation of multiple metal cations with varying ionic radii and coordination preferences induces significant local steric strain. This strain modulates the lattice conformation, influencing the arrangement and flexibility of both the metal–oxygen layers and the intercalated anions, thereby affecting the material’s structural properties and catalytic behavior. FeNiCoCrMn-G represents a typical high-entropy glycerate (HEG) catalyst exhibiting enhanced oxygen evolution reaction (OER) activity due to structural distortion and amorphization [82]. Average atomic strain analysis reveals increasing distortion with the introduction of Co and Mn, and near-complete amorphization upon Cr incorporation. This leads to expanded interlayer spacing in the layered structure, resembling that of anion-intercalated hydroxides, which promotes ion diffusion and intermediate accommodation. The flexible coordination environment allows glycerate anions to adjust bonding modes, facilitating the formation of *OOH species essential for OER.

### High-Entropy Nanocomposites

**Fig. 9:**
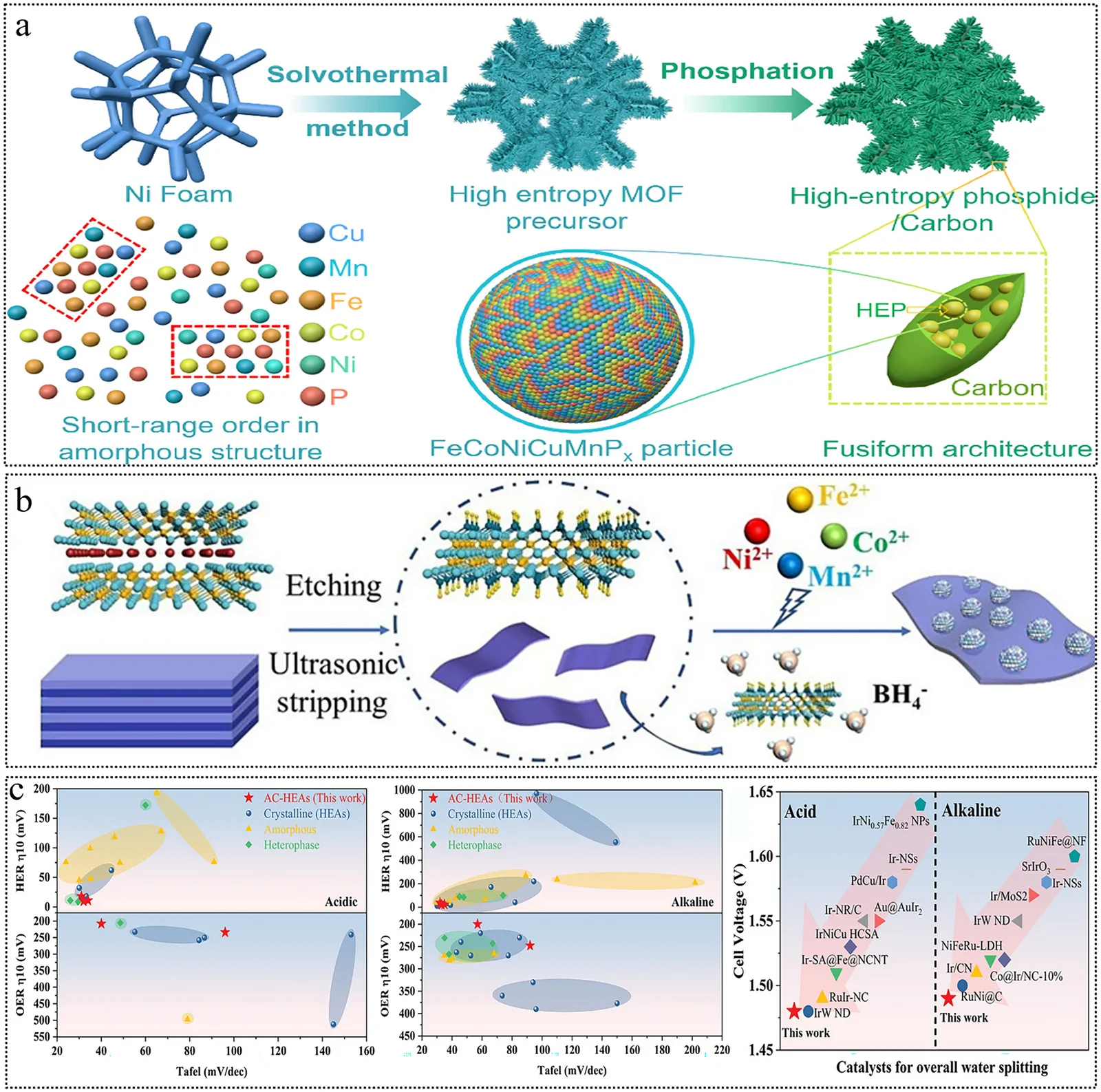
Performance evaluation of HEACs. **a** Schematic representation of preparation of FeCoNiMnCuP_x_/C composite. Reproduced with permission [128]. Copyright 2024, Elsevier. **b** Schematic representation of the HEBO_x_/MXene synthesis process. Reproduced with permission [129]. Copyright 2024, Elsevier. **c** Comparison of catalytic performance of HER, OER, and water splitting in 0.5 M H_2_SO_4_ acidic medium and 1 M KOH alkaline medium. Reproduced with permission [88]. Copyright 2024, John Wiley and Sons

High-entropy amorphous catalysts exhibit abundant active sites and tunable surface chemistry, but often suffer from limited conductivity, aggregation, and structural instability. Constructing nanocomposites by integrating them with conductive supports or functional phases offers an effective solution [147, 148]. For example, carbon frameworks derived from MOFs not only act as conductive scaffolds to accelerate electron transfer but also provide spatial confinement that limits nanoparticle growth, stabilizes amorphous phases, and enhances catalyst dispersion [149, 150]. When high-entropy metal phosphides (e.g., FeCoNiCuMnPx) were encapsulated within carbon layers, the resulting heterostructures exhibited rich active sites (Fig. 9a), short-range ordering, and robust structural stability [128]. These carbon-supported composites (HEM/C) combined the chemical complexity of high-entropy metals with the conductivity and protective nature of carbon, delivering superior OER performance with low overpotentials and long-term operational stability. Metal phosphides such as NiP and FeP can form heterostructures with high-entropy materials, enabling interfacial electronic modulation [119, 122]. The resulting heterojunctions not only tailor the electronic structure of HEA, but also facilitate more efficient charge transfer between the HEA and the phosphide phase, thereby enhancing the overall OER activity of the composite. Additionally, Ti_3_C_2_-MXene was strategically introduced as a conductive support to construct a high-performance nanocomposite electrocatalyst with amorphous high-entropy borate (FeCoNiMnBO_x_) (Fig. 9b) [129]. The resulting FeCoNiMnBO_x_/MXene composite exhibits a low overpotential of 268 mV at 10 mA cm^−2^ and a small Tafel slope of 39.8 mV dec^−1^ in alkaline solution, outperforming both FeCoNiMnBO_x_ alone and commercial RuO_2_ catalysts. The layered Ti_3_C_2_ MXene plays a critical role in preventing the aggregation of FeCoNiMnBO_x_ nanoparticles, thereby increasing the exposure of active sites. More importantly, the strong interfacial interaction between MXene and the amorphous borate enables interfacial charge redistribution and accelerates charge transfer across the interface. This not only enhances the electrical conductivity of the composite but also promotes the oxidation of transition metal species within FeCoNiMnBO_x_, leading to improved OER kinetics.

Beyond conductive support, constructing crystalline-amorphous heterostructures has also emerged as an effective strategy to enhance water-splitting activity. A representative example is the amorphous-crystalline high-entropy alloy (AC-HEA) catalysts synthesized via a facile dealloying strategy from designable multicomponent metallic glass precursors [88]. This method yields nanoporous heterostructured AC-HEAs (e.g., CuAgAuPtPd, CuAgAuIrRu, and CuAgAuPtPdIrRu), composed of nanocrystals (~ 2–3 nm) embedded in an amorphous matrix. Among them, AC-HEA-CuAgAuPtPd exhibited exceptional HER performance, requiring only 9.5 and 20 mV of overpotentials to reach 10 mA cm^−2^ in 0.5 M H_2_SO_4_ and 1.0 M KOH, respectively. AC-HEA-CuAgAuIrRu achieved low overpotentials of 208 mV (acidic) and 200 mV (alkaline) for OER. When assembled into a two-electrode electrolyzer, this bifunctional AC-HEA-CuAgAuIrRu electrode delivers overall water splitting at only 1.48 V in acid and 1.49 V in base at 10 mA cm^−2^, outperforming many state-of-the-art systems (Fig. 9c).

### Practical Water Electrolysis: Opportunities and Challenges

While laboratory studies often use pure water or ideal electrolytes, practical applications must address the challenges posed by complex real-world water sources. These media contain various impurities, dissolved salts, organic compounds, and microorganisms that can significantly impact the efficiency, selectivity, and durability of electrolysis catalysts [151, 152]. For instance, alkaline seawater electrolysis poses greater challenges than pure water electrolysis, primarily due to the presence of microorganisms and the formation of insoluble precipitates such as Mg(OH)_2_ and Ca(OH)_2_, which can poison the catalyst surface and increase the overpotential. Indeed, HEO-FeCoNiMoVO_x_-1.5 exhibited a higher overpotential in alkaline seawater than in pure water [60]. To evaluate its corrosion resistance under these harsher conditions, Tafel corrosion polarization curves were obtained for different samples in alkaline seawater. HEO-FeCoNiMoVO_x_-1.5 delivered the lowest corrosion current density (2.35 μA cm^−2^) and the highest corrosion potential (0.923 V vs. RHE) among all samples, indicating superior anti-corrosion performance. To exclude interference from chloride oxidation producing hypochlorite, long-term stability tests were conducted, followed by detection using N,N-diethyl-1,4-phenylenediamine (DPD) combined with UV–Vis spectroscopy. The results showed that the solution remained colorless and transparent, with no characteristic absorption peaks, indicating no formation of hypochlorite. According to the Pearson hard-soft acid–base (HSAB) principle, HEO-FeCoNiMoVO_x_-1.5 contained a higher proportion of high-valence metal species (as confirmed by XPS), which imparted stronger Lewis acidity. This enabled preferential adsorption of OH^−^ over Cl^−^, effectively preventing chloride oxidation interference and enhancing OER selectivity in alkaline seawater. Finally, to assess the structural stability, post-OER characterizations were performed on the spent catalyst after a 100-h stability test in alkaline seawater. SEM images showed that the microflower morphology of HEO-FeCoNiMoVO_x_-1.5 remained intact on the Ni 3D scaffold without visible corrosion. TEM observations further confirmed the preservation of the amorphous structure without lattice collapse. These results collectively demonstrated that HEO-FeCoNiMoVO_x_-1.5 combined high catalytic activity with outstanding corrosion resistance, excellent OER selectivity, and robust structural stability in alkaline seawater electrolysis.

Additionally, HE(Ru, Mo)-MOFs demonstrated excellent catalytic performance even in complex chemical wastewater. In 1.0 M KOH wastewater electrolyte, LSV curves showed low onset potentials and high current densities comparable to those in freshwater, indicating maintained catalytic activity despite impurities. Notably, the catalyst exhibited lower overpotential in wastewater than in freshwater. EIS curves revealed reduced charge-transfer resistance in wastewater, likely due to the presence of various ionic species and organic compounds that enhanced conductivity and facilitated charge transfer. The Tafel slope was also lower than in control catalysts, confirming faster reaction kinetics. HE(Ru, Mo)-MOFs exhibited superior intrinsic activity, with high turnover frequency and mass activity values outperforming controls. Stability tests showed only a slight increase in overpotential after 1000 cycles and a modest 20% activity decrease after extended continuous operation, highlighting excellent durability under harsh conditions. Overall, these features demonstrated that HE(Ru, Mo)-MOFs combined high efficiency, low overpotential, and robust stability, making them promising candidates for practical water-splitting applications in both freshwater and wastewater environments.

## Understanding the Mechanisms Behind High-entropy Amorphous Catalysts for Water-Splitting Performance

### Multimetallic Synergy

**Fig. 10:**
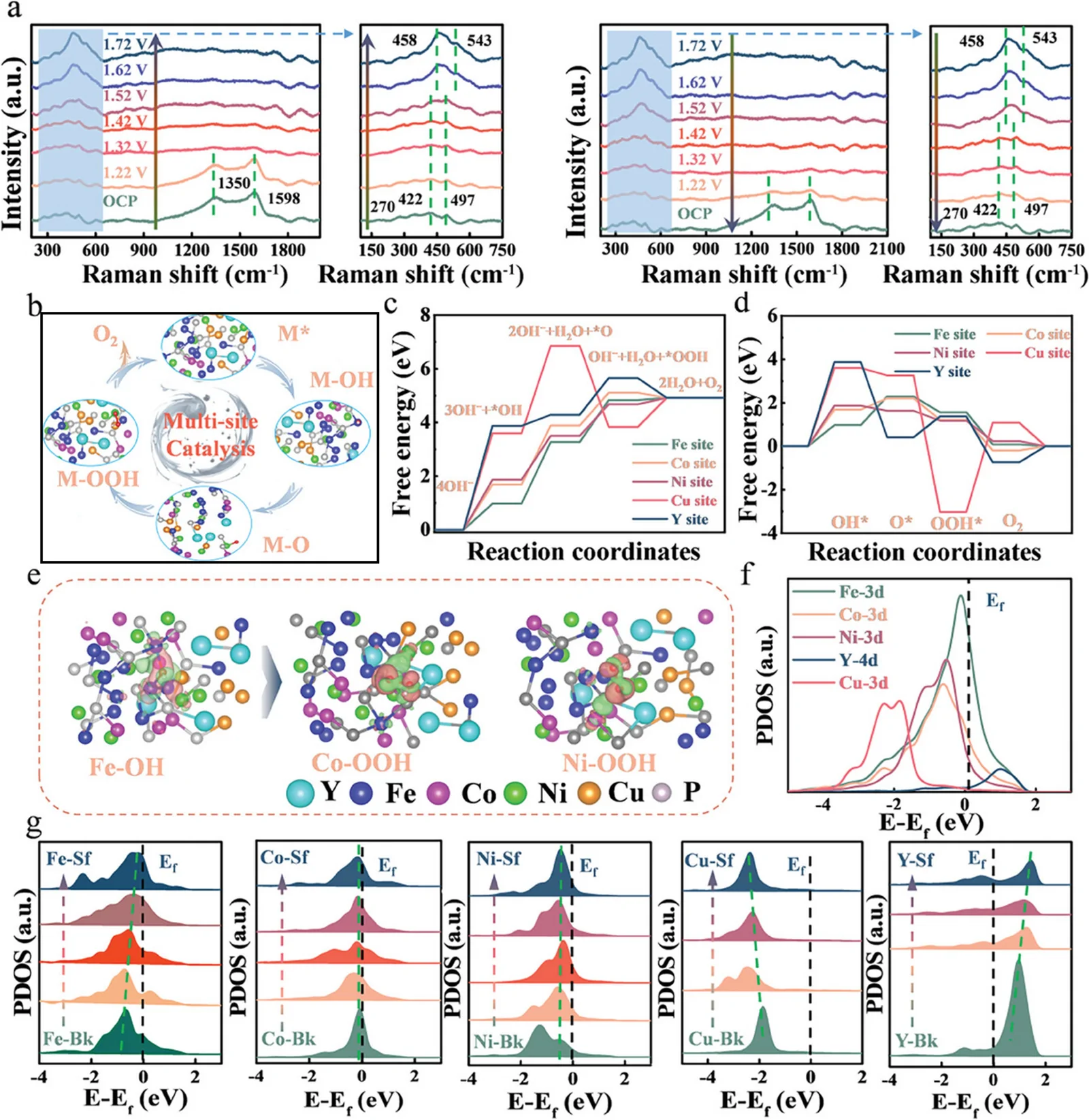
Experiment and calculation data to support multimetallic synergy effect. **a** In-situ Raman spectra of FeCoNiCuYP/C catalysts with positive scan (up arrow) or negative scan (down panel) respectively, and corresponding enlarged view of Raman spectra. **b** Schematic illustration of the intermediate steps in OER pathway on FeCoNiCuYP surface. **c** Gibbs free energy diagrams. **d** energy barrier of the five elementary steps of the OER. **e** Differential charge density of Fe─OH, Co─OOH, Ni─OOH intermediate during OER** f** PDOS of the d orbitals for Fe, Co, Ni, Cu, and Y in FeCoNiCuYP. **g** Site-dependent PDOSs of Fe, Co, Ni, Cu, and Y in HEP. Reproduced with permission [153]. Copyright 2024, John Wiley and Sons

To elucidate the role of multimetallic synergy in promoting OER activity, in-situ Raman, density functional theory (DFT) calculations and ab initio molecular dynamics (AIMD) simulations were performed on a representative high-entropy catalyst. In-situ Raman spectroscopy revealed the reversible formation of TMOOH (Fe(Co/Ni)OOH) during OER, with potential-dependent peak shifts indicating surface strain and dynamic phase evolution that facilitated O intermediate generation (Fig. 10a, b) [153]. The OER pathway, involving four proton-coupled electron transfer (PCET) steps (OH^−^ → OH* → O* → OOH* → O_2_), was dissected by calculating Gibbs free energy changes (Δ*G*) at Fe, Co, Ni, Cu, and Y sites (Fig. 10c, d). The potential-determining steps (PDSs) at Fe and Co sites were the OH* → O* conversion, whereas those at Ni and Y sites corresponded to the initial OH* adsorption. The coexistence of chemically distinct centers enabled spatial separation of intermediates, effectively circumventing the conventional OH-OOH scaling relationship [154]. Energy barrier analysis further showed that Fe sites adjacent to Ni or Cu exhibited favorable OH* adsorption, with OH* migrating efficiently to Co–Ni sites for subsequent O* and OOH* formation (Fig. 10e). At the Co–Ni site, the O* adsorption energy was as low as 1.25 eV, indicating its stability in promoting the OH* → O* and O* → OOH* steps. The Δ*G* values for these two steps at Ni sites were only 1.63 and 1.17 eV, respectively, suggesting that the Co–Ni site (site 6) efficiently catalyzed OOH* formation and subsequent O_2_ evolution. In contrast, OH* adsorption at the Fe-Y site (site 3) resulted in overly negative energy, hindering OH* diffusion to Ni sites. Moreover, if OH* remained at the Fe site, the high ΔG of 2.29 eV for the OH* → O* step implied a slower reaction rate. Thus, the Fe site (site 2) acted as a more active center during the OER. The synergistic interaction between Fe and Co/Ni sites was further supported by experimental activity measurements. Notably, the relay catalysis between Fe and Ni/Co sites helped break the conventional scaling relationship between HOO* and HO* intermediates.

To gain deeper insight, DFT calculations were extended to analyze electronic structure and orbital interactions. Projected density of states (PDOS) revealed that Cu-3*d* orbitals, located ~ 2.0 eV below the Fermi level, acted as electron reservoirs, while Fe-3*d* and Co-3*d* states showed strong overlap near the Fermi level, enabling *d-d* orbital hybridization and charge delocalization (Fig. 10f). Y-4*d* orbitals also contributed to the states near E_f_, enhancing electrical conductivity [155]. Site-resolved PDOS indicated a surface enrichment of Fe-3*d* states and reduced oxidation states at the surface. Charge density difference plots showed electron transfer from Fe, Co, Ni, and Cu to surface O species (Fig. 10g), while Cu and Y exhibited enlarged t_₂g_–e_g_ splitting and underfilled d-bands, facilitating orbital hybridization with adsorbates. Co and Ni maintained consistent electronic structures across depths, contributing to catalytic uniformity [156]. Notably, surface-bound O atoms induced cooperative electron back-donation from adjacent Cu, Y, and P atoms, forming a dynamic charge-transfer network. Increased e_g_ filling at Fe and Y sites upon O adsorption suggested strong Fe–O and Y–O bonds, further stabilized by Fe–P interactions [157]. Collectively, these results demonstrated that high-entropy configurations induced spatially adaptive electronic environments, enabling multimetallic synergy that enhances both the thermodynamics and kinetics of OER [16].

### Amorphization Effect

**Fig. 11:**
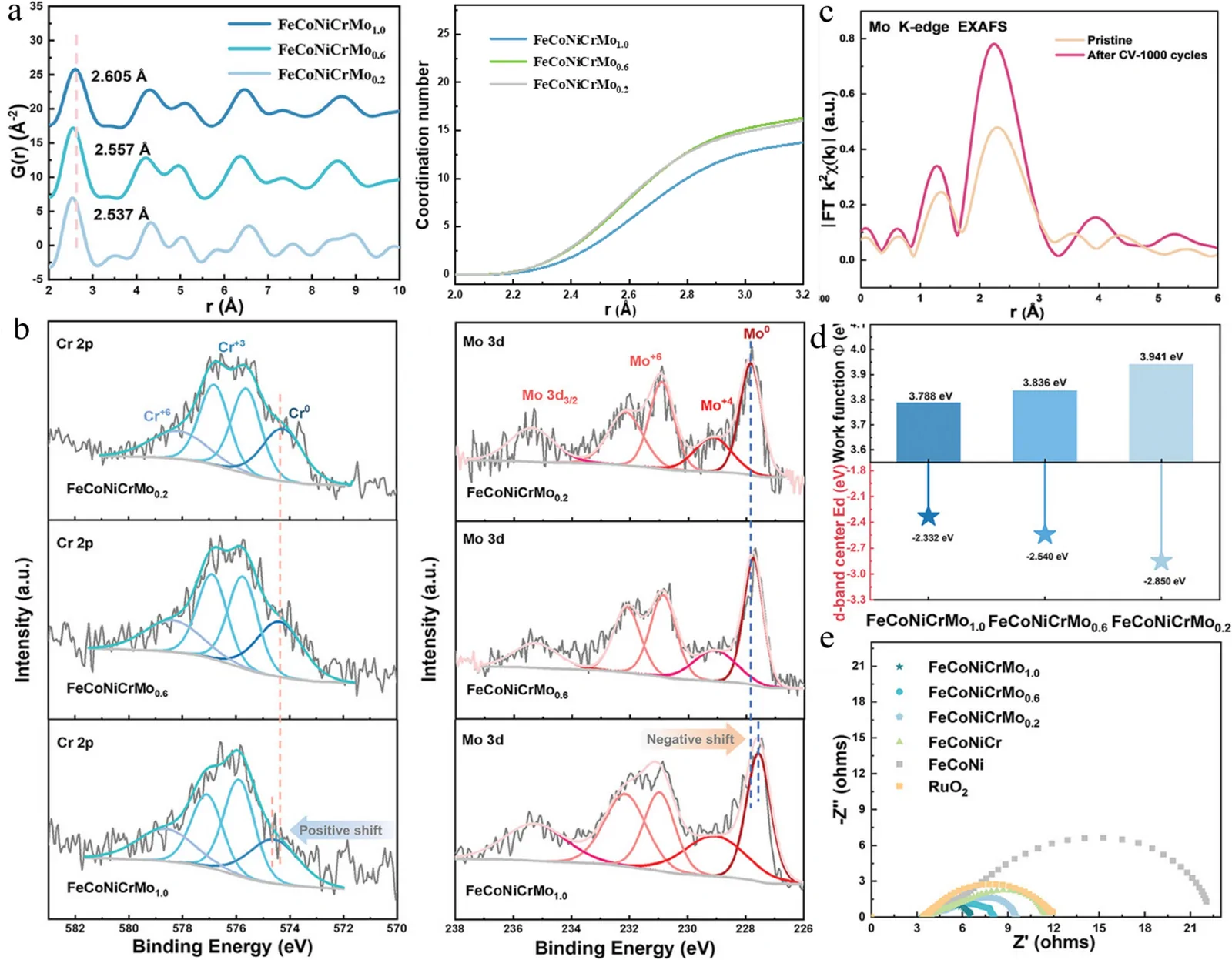
Experiment and simulated data to support structural disorder effect. **a** Reduced pair distribution function *G*(*r*) patterns and the integrated area of the nearest-neighbor coordination shell of the radial distribution function profiles. **b** High-resolution XPS spectra of Cr 2p and Mo 3d. **c** EXAFS patterns of pristine and CV-1000 cycles FeCoNiCrMo_1.0_. **d** Work functions and d-band centers of FeCoNiCrMo_0.2_, FeCoNiCrMo_0.6_, and FeCoNiCrMo_1.0_. **e** Corresponding EIS spectra. Reproduced with permission [159]. Copyright 2024, John Wiley and Sons

Catalytic performance is closely related to the atomic coordination and electronic structure of active sites. A lower atomic coordination number is often accompanied by a looser surface arrangement, a narrower electronic band width, and an upward-shifted d-band center, features that together enhance the adsorption strength of reaction intermediates [158]. To uncover such structure-performance relationships in FeCoNiCrMo_x_ catalysts, a comprehensive characterization and theoretical approach was employed [159]. Pair distribution function (PDF) analysis demonstrated the loose atomic arrangement of FeCoNiCrMo_1.0_ and the smallest nearest-neighbor coordination number (Fig. 11a). High-resolution XPS revealed that FeCoNiCrMo_1.0_ exhibited a distinct positive shift in the Cr 2*p* binding energy and a negative shift in the Mo 3*d* binding energy, indicating electron transfer from Cr to the more electronegative Mo (Fig. 11b). EXAFS analysis at the Mo K-edge further confirmed that after 1000 CV cycles, the Mo coordination peaks intensified and shifted to lower *R*-values, implying a denser packing around Mo due to local structural rearrangements (Fig. 11c). Valence band spectra (VBS) showed that FeCoNiCrMo_1.0_ possessed the smallest work function (3.788 eV), suggesting a lower energy barrier for electron excitation and emission. Increasing Mo content induced an upward shift of the d-band center, with FeCoNiCrMo_1.0_ reaching − 2.332 eV (Fig. 11d). This upshift arised from the tensile strain (~ 2.61%) induced by Mo incorporation, which decreased orbital overlap, narrowed the band width, and destabilized the d electrons, thereby increasing their reactivity. An elevated d-band center enhanced the adsorption of oxygen-containing intermediates (O, OH, OOH), facilitating their activation and turnover. Such Mo-Cr interactions facilitated electron delivery during OER, consistent with the conductivity trend (FeCoNiCrMo_1.0_ > FeCoNiCrMo_0.6_ > FeCoNiCrMo_0.2_) (Fig. 11e).

**Fig. 12:**
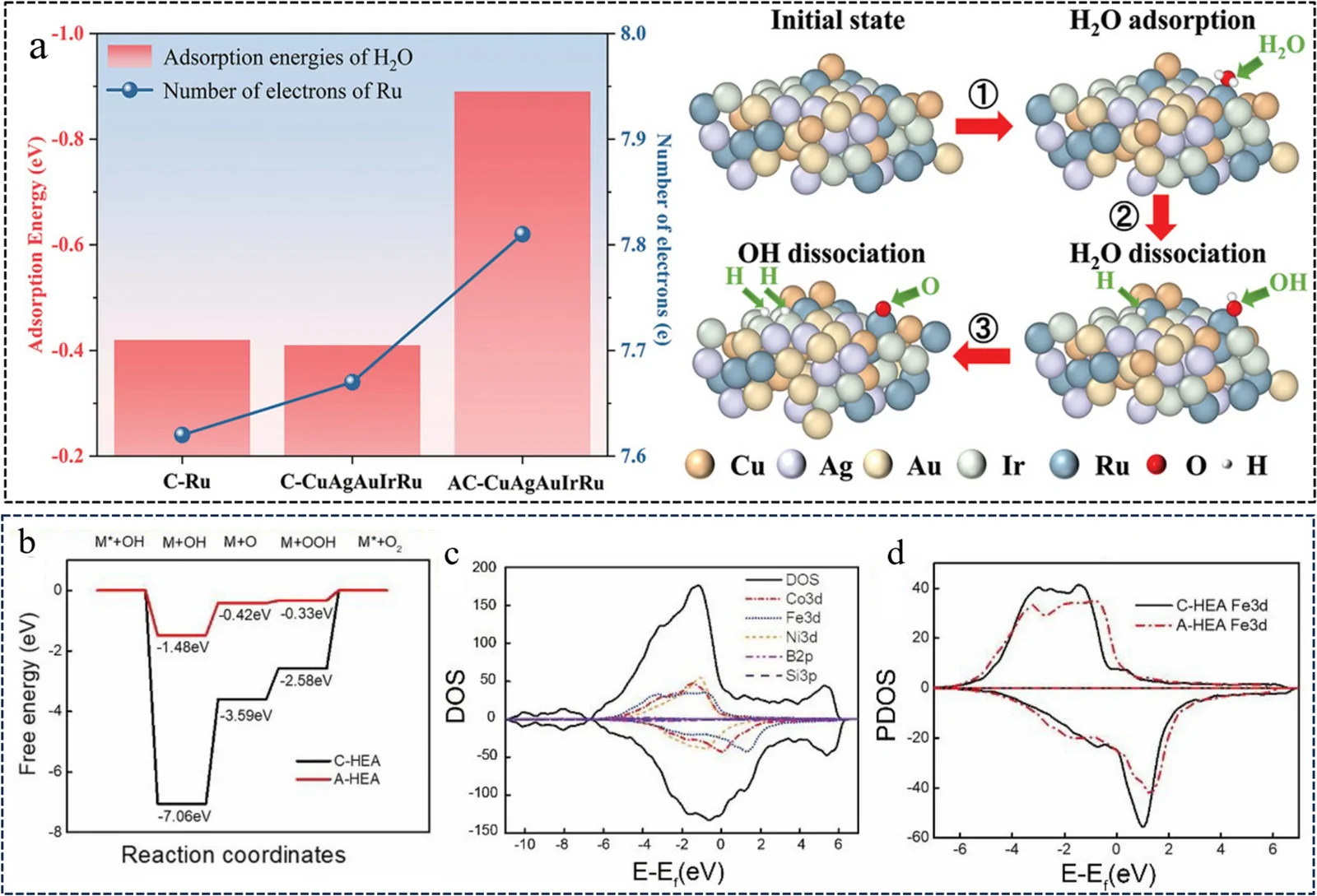
DFT calculation data. **a** Calculated adsorption free energy and number of electrons for H_2_O adsorption at the equilibrium potential for C-Ru, C-HEA-CuAgAuIrRu and AC-HEA-CuAgAuIrRu catalysts and schematic representation of the amorphous surface atomic configuration of AC-HEA-CuAgAuIrRu during water molecule adsorption and dissociation. Reproduced with permission [88]. Copyright 2024, John Wiley and Sons. **b** Free energy diagrams of OER over the A-HEA and C-HEA slab catalysts. **c** Density of states of A-HEA. **d** Partial density of states of Fe. Reproduced with permission [121]. Copyright 2021, Elsevier

Additionally, a model comparison between crystalline Ru (C-Ru), crystalline HEA (C-HEA: CuAgAuIrRu), and amorphous HEA (AC-HEA) demonstrated that the random atomic distribution in AC-HEA generated abundant defect sites and coordinatively unsaturated centers, producing strong d-band ligand effects [88]. Density functional theory (DFT) calculations showed significantly stronger water adsorption on AC-HEA (Ru–O bond length 2.27 Å; adsorption energy − 0.89 eV) than on C-Ru (− 0.42 eV) or C-HEA (− 0.41 eV), attributed to higher local electron density at Ru sites (Fig. 12a). For crystalline Fe_29_Co_27_Ni_23_Si_9_B_12_ (C-HEA), the largest energy barrier (− 7.06 eV) occurred at OH adsorption—the rate-determining step—while in the amorphous counterpart (A-HEA), this barrier was dramatically reduced to − 1.48 eV, approaching thermoneutrality (Fig. 12b) [121]. Bader charge analysis indicated weaker OH binding in A-HEA (Δ*E*_OH_ = − 1.78 eV) than in C-HEA (− 3.77 eV), suggesting optimized adsorption free energy. Overall, amorphization tunes the electronic structure by enhancing defect density, modulating the d-band center, and optimizing intermediate adsorption, thereby lowering the activation barriers and boosting OER activity (Fig. 12c, d).

### In-Situ Reconstruction

**Fig. 13:**
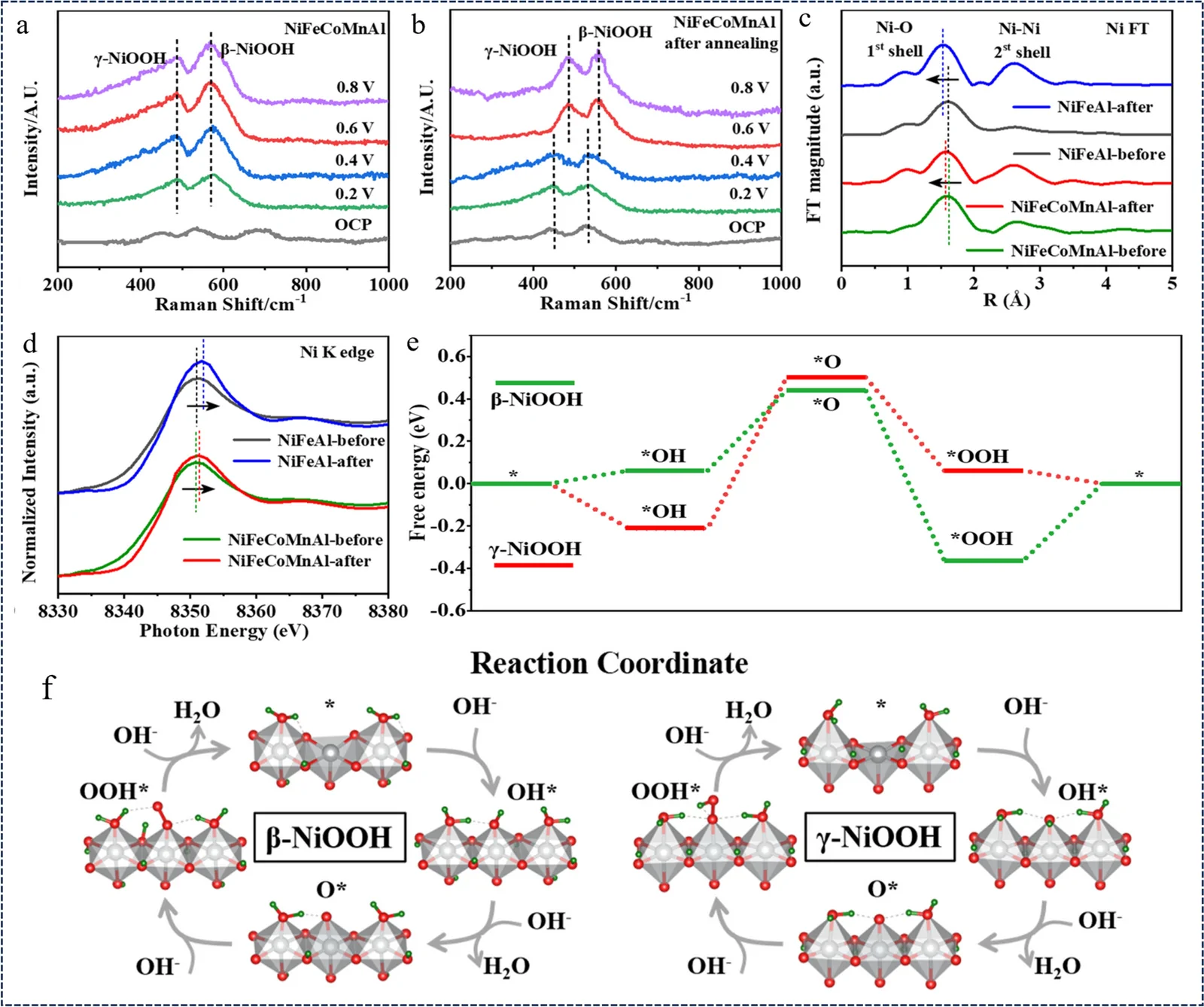
Experiment and calculation data to support in-situ reconstruction. The *operando* Raman spectra of **a** the NiFeCoMnAl and **b** NiFeCoMnAl-after annealing catalysts under various applied potentials. **c** FT-EXAFS and **d** XANES of NiFeAl and NiFeCoMnAl catalysts before and after OER at Ni K-edge. **e** Reaction energy diagram and **f** schematic presentation of water oxidation on *β*-NiOOH and *γ*-NiOOH models at different stages of the reaction. Reproduced with permission[111]. Copyright 2022, Elsevier

Due to the metastable nature of the amorphous phase and the lattice distortion effects inherent to high-entropy catalysts, high-entropy amorphous catalysts are more prone to reconstruction under electrochemical conditions. This reconstruction enhances the catalyst’s stability while preserving its intrinsic activity. Employing advanced characterization techniques to identify such reconstruction can provide deeper insights into the origins of catalyst activity and durability. Operando Raman spectroscopy revealed the dynamic reconstruction behavior of Ni-based active phases during the OER process (Fig. 13a) [111]. At open-circuit potential (OCP), NiFeCoMnAl exhibited a pronounced Ni–O stretching vibration peak at ~ 680 cm^−1^, corresponding to hydroxide phases, indicating a higher initial fraction of hydroxide structures, consistent with O1*s* XPS results. Upon increasing the applied potential, the doublet Ni–O peaks of the oxide phase gradually red-shifted, accompanied by the emergence of characteristic peaks of *β*-NiOOH (~ 555 cm^−1^) and *γ*-NiOOH (~ 475 cm^−1^), confirming the in-situ transformation of Ni sites into NiOOH active phases. Notably, Mn-doped NiFeCoMnAl predominantly formed *β*-NiOOH, whereas undoped NiFeAl favored *γ*-NiOOH. This difference can be attributed to the electron-rich environment provided by Mn, which lowered the average oxidation state of Ni and facilitated the formation of *β*-NiOOH intermediates with Ni ≤  + 3. Compared with the crystalline counterpart, amorphous NiFeCoMnAl fully transformed into the NiOOH phase at a low potential of 0.2 V, while the crystalline phase required 0.6 V to initiate NiOOH formation, demonstrating the amorphous structure’s advantage in promoting active-phase generation (Fig. 13b). XAS measurements further verified this reconstruction process (Fig. 13c, d). Before OER, the Ni K-edge white line positions of NiFeCoMnAl and NiFeAl were nearly identical, and the EXAFS peaks at ~ 1.5 and ~ 2.6 Å were assigned to Ni–O and Ni–Ni coordination, respectively. After OER, the Ni K-edge shifted toward higher energies, indicating oxidation of Ni; the smaller shift in NiFeCoMnAl compared to NiFeAl suggests that Mn doping suppressed overoxidation and maintained lower Ni valence states, consistent with XPS analysis. In the EXAFS spectra, both Ni–O and Ni–Ni peaks shifted slightly toward lower *R*, with a more pronounced shift for NiFeAl, implying that Mn-doped samples retained longer Ni–O bonds and more hydroxide-like structural features. These results corroborated that Mn doping promotes the preferential formation and stabilization of *β*-NiOOH active phases. DFT calculations further supported this conclusion (Fig. 13e). The free energy profiles for *β*-NiOOH and *γ*-NiOOH surfaces showed that O* formation was the potential-limiting step for both, but *β*-NiOOH exhibited a much lower overpotential (0.38 eV) than *γ*-NiOOH (0.71 eV). This indicated that *β*-NiOOH was thermodynamically more favorable for driving OER and optimized the adsorption of *OH and *O intermediate. In summary, operando Raman, XAS, and DFT results collectively elucidate the in-situ reconstruction mechanism of Ni-based catalysts during OER: Mn doping tunes the Ni valence state, enabling amorphous NiFeCoMnAl to preferentially generate highly active *β*-NiOOH phases at low potentials, thereby significantly enhancing OER performance.

## Conclusions and Future Perspectives

In summary, the development of high-entropy catalysts (HECs) has demonstrated significant progress in both synthetic methodology and electrocatalytic performance. The adopted synthesis strategies not only enable the formation of amorphous structures with tailored compositions and local environments, but also offer distinct advantages such as high tunability and low energy consumption. These catalysts have exhibited exceptional activity and durability across a broad pH range—including acidic, neutral, and alkaline media—as well as in challenging conditions such as seawater electrolysis and industrial wastewater treatment. Notably, in-situ characterization techniques reveal that structural reconstruction occurs during electrolysis, resulting in the formation of crystalline oxyhydroxides as the true active phases. This transformation not only generates new catalytically active species but also preserves the high structural stability of the catalyst, thereby contributing to both enhanced activity and durability. Collectively, these findings position high-entropy amorphous catalysts as a versatile and robust platform for next-generation water-splitting technologies. To further advance this promising field, several future directions merit consideration:

### Accelerating Electrocatalyst Design Through DFT-Machine Learning Integration

**Fig. 14:**
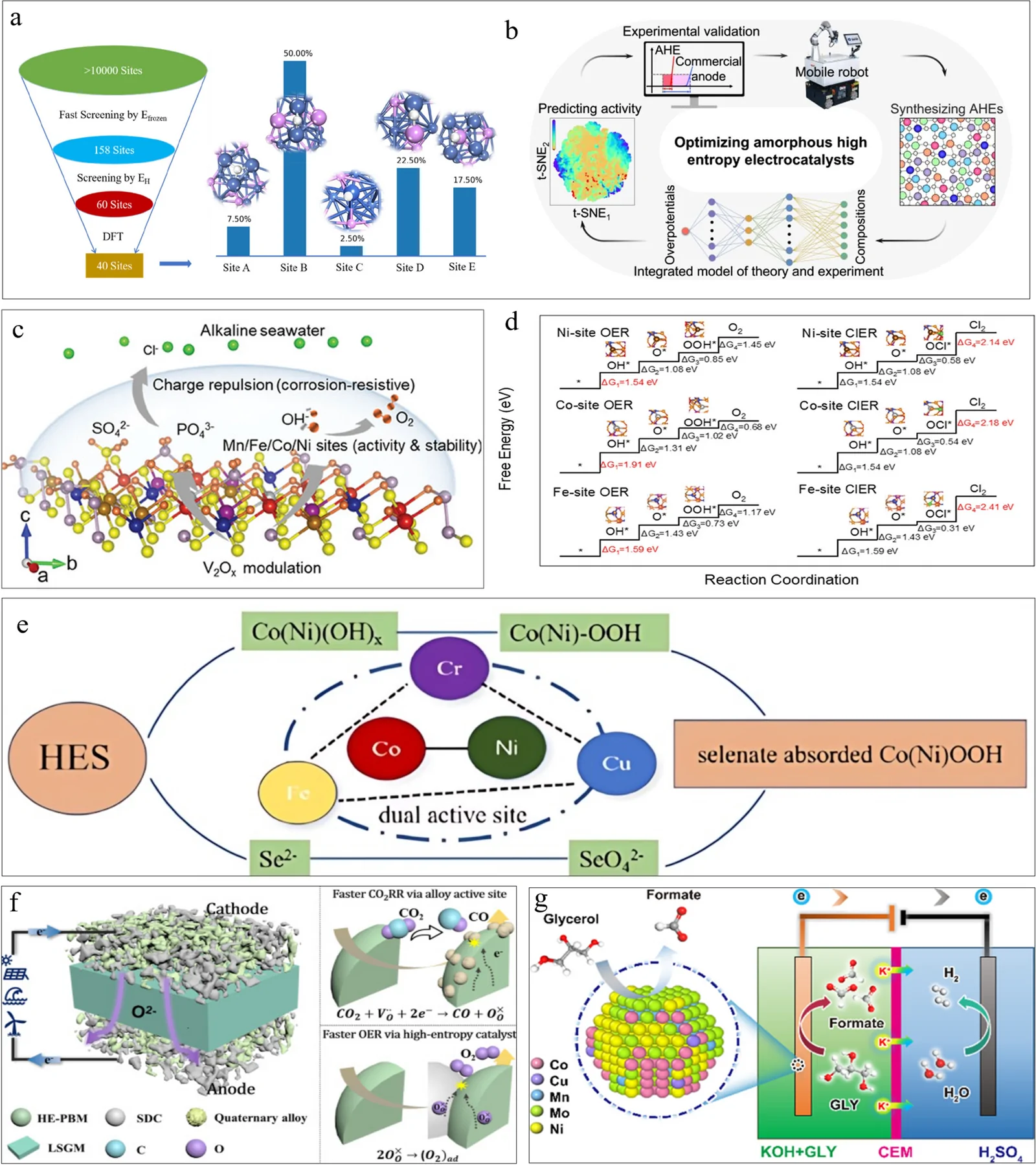
**a** High-throughput screening process and results using predictive model. Reproduced with permission [160]. Copyright 2020, American Chemical Society. **b** Schematic illustration of automatic discovery and optimal generation of amorphous high-entropy electrocatalysts. Reproduced with permission [161]. Copyright 2025, American Chemical Society. **c** Schematic diagram of reconstructed HEPS modulated chlorine resistance and metal active sites to boost efficient and durable seawater electrolysis. Mn (purple), Fe (blue), Co (gray), Ni (brown), V (red), P (red), S (yellow), O (orange), H (white) and Cl (green). **d** OER and ClER mechanism. Reproduced with permission [162]. Copyright 2025, John Wiley and Sons. **e** Proposed mechanism of electrochemical reconstruction during OER process. Reproduced with permission [163]. Copyright 2023, Elsevier. **f** Schematic of electrolysis system of CO_2_RR and OER [165]. **g** Schematic of electrolysis system of GOR and HER. Reproduced with permission [166]. Copyright 2022, American Chemistry Society

The prediction of chemisorption energies is central to high-throughput screening of active electrocatalysts yet remains a formidable challenge—particularly for amorphous materials whose disordered atomic arrangements and dynamically evolving surface states hinder conventional structure–property correlations. Although amorphous catalysts often exhibit superior activity water splitting, predictive models for their adsorption energetics are scarce and unreliable. This difficulty is exemplified by the limited efficiency of advanced symmetry function-based neural networks in predicting adsorption energies on amorphous surfaces, where flexible atomic configurations undergo significant relaxation upon adsorption. A promising future direction lies in the integration of DFT calculations, genetic algorithm-based structural searches, and advanced machine learning (ML) models into a stepwise predictive framework. The study of Zhang et al. proposed a strategy toward a deep understanding and an accurate prediction for the adsorption energy of amorphous surfaces (Fig. 14a) [160]. The strategy split the complicated adsorption energy prediction on amorphous catalysts into two subtasks: the frozen adsorption energy (E_frozen_) and the structural relaxation energy (E_relax_). Furthermore, they proposed an atomic expansion method which achieved the prediction of H adsorption energy (E_H_ = E_relax_ + E_frozen_) at a high accuracy (RMSE < 0.1 eV). Subsequently, they proposed a predictive mode to explore active sites on amorphous catalysts for HER. The combination of machine learning and DFT calculations improved the accuracy of active-site identification.

Lei et al. further established a composition-activity relationship using ML techniques and identified an optimal composition group by exploring the entire design space of over 1,900,000 compositions (Fig. 14b) [161]. The ML model was validated with 100 compositions from the high-activity region and 588 from the low-activity region, achieving nearly 100% recall. The predicted optimal amorphous high-entropy electrocatalyst exhibited an ultralow overpotential of 159 mV at 10 mA cm^−2^ for the alkaline OER in 1 M KOH and showed exceptional durability of 10,218 h under a practical current density of 1 A cm^−2^ in 6 M KOH. This work provided a general strategy for the automatic discovery and optimization of amorphous high-entropy oxyhydroxide electrocatalysts and had the potential to significantly influence the development of other amorphous high-entropy materials.

### Exploring the Hidden Potential of Amorphous High-entropy Non-oxide Anionic Catalysts for Seawater Electrolysis

Although amorphous high-entropy catalysts containing non-oxide anions (e.g., P^3−^, S^2−^, B^3−^) have been developed, their structural evolution and reaction mechanisms remain insufficiently explored. These materials exhibit unique disordered configurations that enable dynamic surface reconstruction during electrochemical operation. Notably, in seawater electrolysis, they can in-situ generate anion-derived protective layers—such as phosphates or borates—that effectively suppress chloride oxidation while sustaining oxygen evolution activity. This self-adaptive behavior offers a promising pathway to address the stability challenges posed by Cl^−^ in saline environments. To fully realize their potential, future research should focus on in-situ/operando characterization of surface evolution, DFT-guided investigation of anion-metal interactions, and rational design of entropy-stabilized non-oxide frameworks. As a representative example, a high-entropy sulfur phosphide catalyst, MnFeCoNiVPS_3_, was strategically designed based on the concept of spatially separated functional regions to address the challenges of seawater electrolysis [162]. This high-entropy phosphorus sulfide (HEPS) exhibited excellent OER performance in 1 M KOH seawater, requiring only 245 and 313 mV overpotentials to reach current densities of 10 and 100 mA cm^−2^, respectively. Combined experimental and theoretical investigations revealed that the remarkable stability of HEPS was attributed to the formation of negatively charged SO_4_^2−^ and PO_4_^3−^ surface species, which selectively repelled Cl^−^ ions and protected the metal active sites from corrosion and aggregation (Fig. 14c, d). Moreover, multivalent V_2_O_x_ species effectively modulated the electronic structures of Mn, Fe, Co, and Ni, preventing their overoxidation and reducing metal dissolution.

### Constructing In-Situ Amorphization-Driven Interface in High-Entropy Catalysts for Enhanced Electrocatalysis

Studies have shown that high-entropy amorphous catalysts undergo structural reconstruction during electrocatalysis, transforming into amorphous-crystalline core–shell structures, which significantly enhances their catalytic performance. Accordingly, in-situ amorphization of high-entropy crystalline catalysts can serve as a promising design strategy. Specifically, partial in-situ transformation of the crystalline phase into an amorphous structure leads to the formation of crystalline-amorphous hetero architectures, offering dynamic active interfaces for improved catalytic activity. Strategies to induce such transformations include voltage-driven phase transitions, surface etching, ion leaching, and redox-induced bond rearrangement. For example, a flower-like high-entropy selenide, (CoNiFeCuCr)Se (F-HES), was synthesized via a two-step solvothermal method [163]. Post-electrolysis characterizations revealed that the catalyst surface underwent amorphization due to Cr dissolution, forming a mixed structure composed of metal (oxy)hydroxides and selenates, while the inner core retained its crystalline framework. DFT calculations indicated that the high-entropy coordination environment, coupled with the presence of surface-adsorbed selenate species, enhanced the inherent reactivity of the active sites and accelerated the reaction kinetics (Fig. 14e). Zhang et al. employed CV activation to trigger the electrochemical reconstruction of FeCoNiMnZnMoOx-1.0/NF into amorphous FeCoNiMnZnMoOOH/NF [164]. Over 100 CV cycles in strong alkaline solution, Zn and Mo underwent preferential dissolution, generating [Zn(OH)_4_]^2−^ and MoO_4_^2−^ species, while Fe, Co, Ni, and Mn were oxidatively hydroxylated. This selective etching created abundant oxygen vacancies (O_v_) and followed a dissolution-redeposition pathway: part of the dissolved Zn and Mo diffused back into the residual framework, driven by a localized pH drop from continuous OH⁻ consumption at the catalyst-electrolyte interface. The redeposition of these species, coupled with O_2_ desorption and dynamic O_v_ replenishment, guided the complete transformation from FeCoNiMnZnMoOx to FeCoNiMnZnMoOOH.

### Coupling HER/OER with Value-Added Redox Reactions for Integrated Electrochemical Systems

In traditional water electrolysis, the HER and OER serve as the cathodic and anodic half-reactions, respectively. However, the OER suffers from sluggish kinetics and yields low-value O_2_, while HER often relies on energy-intensive operation. To address these limitations, a growing strategy involves coupling HER or OER with alternative oxidation or reduction reactions that not only lower overall energy consumption but also produce value-added products. When applying high-entropy amorphous catalysts in such coupling systems, techno-economic analysis (TEA) should be conducted to assess the balance between system inputs and outputs, thereby exploring the practical application potential of these catalysts. An innovative symmetrical electrode based on high-entropy perovskite, formulated as Pr_0.5_Ba_0.5_Mn_0.2_Fe_0.2_Co_0.2_Ni_0.2_Cu_0.2_O_3-δ_ (HE-PBM), has exhibited outstanding bifunctional catalytic activity for both the OER and carbon dioxide reduction reaction (CO_2_RR) (Fig. 14f) [165]. The incorporation of multiple transition metals (Mn, Fe, Co, Ni, and Cu) at the B-site of the perovskite structure significantly enhanced OER kinetics at the anode. Under reducing conditions, in-situ generation of Fe–Co–Ni–Cu alloy nanoparticles from the HE-PBM cathode contributed to its high CO_2_RR activity. Additionally, Fan et al. developed a new class of self-supported HEA electrodes by uniformly growing HEA nanoparticles on carbon cloth substrates [166]. Among them, the HEA-CoNiCuMnMo electrode displayed excellent performance for glucose oxidation reaction (GOR), featuring a low overpotential and high selectivity toward formate production. To understand the origin of its catalytic activity, a custom machine learning-assisted Monte Carlo simulation was employed to identify Mo sites—coordinated by Mn, Mo, and Ni—as the key active centers. Furthermore, a hybrid flow electrolyzer was assembled by coupling alkaline GOR at the anode (using HEA-CoNiCuMnMo) with acidic hydrogen evolution at the cathode (using commercial RhIr/Ti). This system achieved a current density of 10 mA cm^−2^ at a low voltage of just 0.55 V, and sustained stable operation for over 12 days at 50 mA cm^−2^, maintaining Faradaic efficiencies exceeding 99% for hydrogen and 92% for formate generation (Fig. 14g).

## Data Availability

No primary research results, software, or codes have been included, and no new data were generated or analyzed as part of this review.
